# Dual‐Strategy of Cation‐Doping and Nanoengineering Enables Fast and Stable Sodium‐Ion Storage in a Novel Fe/Mn‐Based Layered Oxide Cathode

**DOI:** 10.1002/advs.202002199

**Published:** 2020-09-24

**Authors:** Qiuyu Shen, Xudong Zhao, Yongchang Liu, Youpeng Li, Jian Zhang, Ning Zhang, Chenghao Yang, Jun Chen

**Affiliations:** ^1^ Beijing Advanced Innovation Center for Materials Genome Engineering Institute for Advanced Materials and Technology State Key Laboratory for Advanced Metals and Materials University of Science and Technology Beijing Beijing 100083 China; ^2^ Key Laboratory of Advanced Energy Materials Chemistry (Ministry of Education) Nankai University Tianjin 300071 China; ^3^ New Energy Research Institute School of Environment and Energy South China University of Technology Guangzhou 510006 China; ^4^ Beijing Advanced Innovation Center for Materials Genome Engineering School of Mathematics and Physics University of Science and Technology Beijing Beijing 100083 China; ^5^ College of Chemistry & Environmental Science Hebei University Baoding 071002 China

**Keywords:** electrospinning, Fe/Mn‐based layered oxide cathodes, nanoengineering, nanostructures, reaction mechanisms, sodium‐ion batteries

## Abstract

Iron/manganese‐based layered transition metal oxides have risen to prominence as prospective cathodes for sodium‐ion batteries (SIBs) owing to their abundant resources and high theoretical specific capacities, yet they still suffer from rapid capacity fading. Herein, a dual‐strategy is developed to boost the Na‐storage performance of the Fe/Mn‐based layered oxide cathode by copper (Cu) doping and nanoengineering. The P2‐Na_0.76_Cu_0.22_Fe_0.30_Mn_0.48_O_2_ cathode material synthesized by electrospinning exhibits the pearl necklace‐like hierarchical nanostructures assembled by nanograins with sizes of 50–150 nm. The synergistic effects of Cu doping and nanotechnology enable high Na^+^ coefficients and low ionic migration energy barrier, as well as highly reversible structure evolution and Cu/Fe/Mn valence variation upon repeated sodium insertion/extraction; thus, the P2‐Na_0.76_Cu_0.22_Fe_0.30_Mn_0.48_O_2_ nano‐necklaces yield fabulous rate capability (125.4 mA h g^−1^ at 0.1 C with 56.5 mA h g^−1^ at 20 C) and excellent cyclic stability (≈79% capacity retention after 300 cycles). Additionally, a promising energy density of 177.4 Wh kg^−1^ is demonstrated in a prototype soft‐package Na‐ion full battery constructed by the tailored nano‐necklaces cathode and hard carbon anode. This work symbolizes a step forward in the development of Fe/Mn‐based layered oxides as high‐performance cathodes for SIBs.

## Graphical abstract

Pearl necklace‐like hierarchical nanostructures of a P2‐Na_0.76_Cu_0.22_Fe_0.30_Mn_0.48_O_2_ cathode are synthesized by electrospinning and evaluated in sodium‐ion batteries. The synergistic effects of Cu doping and nanoengineering enable high Na^+^ coefficients and low ionic migration energy barrier, as well as highly reversible structure evolution and Cu/Fe/Mn valence variation upon repeated sodium insertion/extraction, rendering fabulous rate capability and excellent cyclic stability.

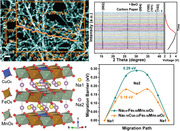

## 1 Introduction

Realizing a secure and sustainable energy supply sets a high standard for large‐scale energy storage systems, which requires the systematic utilization of renewable energy sources and thus facilitates the development of rechargeable batteries.^[^ ^1^ ^]^ Recently, researches on sodium‐ion batteries (SIBs) continuously surge owing to the inexhaustible supply and wide distribution of sodium resources compared with lithium, and the similar electrochemistry to that of lithium‐ion batteries. These advantages pave the way for SIBs to be a forthcoming candidate for grid‐scale energy storage applications.^[^ ^2^, ^3^, ^4^ ^]^ Nonetheless, the larger size in conjunction with the heavier mass of Na^+^ versus Li^+^ commonly leads to undesirable sluggish reaction kinetics and even severe structural collapse of Na‐storage electrode materials, hindering this technology to reach a commercial prosperity.^[^ ^5^, ^6^ ^]^ Given that cathode is the crucial element dominating the cost and electrochemical performance of batteries, great efforts should be devoted to exploring advanced cathode materials with high redox potential, high capacity, and rapid sodium‐ion mobility.^[^ ^7^, ^8^ ^]^ Among the various reported cathode candidates, such as transition metal oxides (TMOs),^[^ ^9^, ^10^, ^11^, ^12^, ^13^, ^14^, ^15^, ^16^ ^]^ polyanion‐type compounds,^[^ ^17^, ^18^, ^19^, ^20^, ^21^, ^22^ ^]^ Prussian blue analogs,^[^ ^23^ ^]^ and organic salts,^[^ ^24^ ^]^ layered TMOs have gained considerable attention on account of their high theoretical capacities and special 2D structural merits.^[^ ^9^, ^13^, ^25^ ^]^ Typically, the layered TMOs can be mainly divided into two polymorphs: P2 type (P: prismatic) and O3 type (O: octahedral) in accordance with the coordination environment of Na ions, where the “2” or “3” means the number of transition metal layers in a single cell unit.^[^ ^26^ ^]^ The P2‐type oxides featuring large prismatic residing sites and facile diffusion paths for sodium ions generally exhibit better electrochemical properties than the O3‐type materials.^[^ ^27^, ^28^, ^29^, ^30^ ^]^ For instance, P2‐Na_2/3_Fe_1/2_Mn_1/2_O_2_ is recognized to be an appealing cathode for SIBs in view of its abundance and low cost in raw materials, environmental benignity, and remarkable theoretical specific capacity (175 mA h g^−1^).^[^ ^28^, ^31^, ^32^ ^]^ However, deteriorated cycling performance is usually observed for this material due to the Jahn–Teller lattice distortions caused by Mn^3+^ and the phase transitions at high voltage during Na^+^ (de)intercalation.^[^ ^33^, ^34^, ^35^, ^36^ ^]^

Aiming at addressing the aforementioned issues facing the layered Na‐Fe‐Mn‐O system, a variety of strategies have been attempted to achieve better electrochemical performance, such as metallic cations (Ti^4+^, Cu^2+^, Co^3+^, etc.) doping to stabilize the P2 structure,^[^ ^25^, ^35^, ^37^, ^38^, ^39^, ^40^, ^41^, ^42^ ^]^ surface engineering to retard the detrimental side reactions,^[^ ^43^, ^44^ ^]^ or nanosizing to shorten the ionic diffusion paths.^[^ ^45^ ^]^ For example, Rojo's group successfully synthesized a Ti‐substituted P2‐Na_2/3_Mn_0.8_Fe_0.1_Ti_0.1_O_2_ material, which turned out to proceed with a solid solution reaction rather than the P2‐O2 phase transition during the charge/discharge process and showed a long cycling life of 87% capacity retention after 300 cycles.^[^ ^25^ ^]^ Hu and co‐workers fabricated a free‐standing electrode of P2‐Na_2/3_Fe_1/2_Mn_1/2_O_2_ with graphene flakes, in which the 2D graphene effectively improved the reaction activity of the oxide particles by forming a 3D conductive network, delivering a fascinating discharge capacity of 156 mA h g^−1^ at 0.1 C with a capacity retention of 61% after 40 cycles.^[^ ^43^ ^]^ Guo's team focused on the morphology design and the tailored P2‐Na_2/3_Fe_1/2_Mn_1/2_O_2_ nanofibers enabled an optimized rate performance of 142 mA h g^−1^ and 52 mA h g^−1^ at 1 C and 15 C, respectively.^[^ ^45^ ^]^ Despite inspiring progresses have been made, in terms of practical applications, it is still not competent to well combine good cyclic stability with superior rate capability in P2‐Na_2/3_Fe_1/2_Mn_1/2_O_2_ only through a single modification route. Considering that cations doping can stabilize the crystal structure and nanoengineering can facilitate the reaction kinetics, the incorporation of the aforementioned two strategies may offer an opportunity to simultaneously boost the fast and stable sodium‐ion storage in a Fe/Mn‐based layered oxide cathode. However, to the best of our knowledge, such a dual‐strategy has scarcely been adopted which still remains a big challenge to date.

In the light of the foregoing, we herein successfully synthesized the pearl necklace‐like hierarchical nanostructures of a novel P2‐Na_0.76_Cu_0.22_Fe_0.30_Mn_0.48_O_2_ cathode through electrospinning and fully dug out the effects of Cu^2+^ doping on electrochemical properties. Such a distinctive nano‐necklace architecture assembled by secondary nanograins (50–150 nm, the smallest size ever reached for Fe/Mn‐based layered oxides) can expose rich active sites readily accessible to electrolyte, shorten the ionic diffusion distance, and alleviate the nanoparticles from aggregation upon repeated Na^+^ insertion/extraction. Meanwhile, the Cu substitution of Fe is proved to accelerate the Na^+^ transportation and restrain the Na^+^/vacancy ordering during cycling. Consequently, outstanding rate capability (125.4 mA h g^−1^ at 0.1 C and 56.5 mA h g^−1^ at 20 C) and excellent cycling stability (≈79% capacity retention after 300 cycles) between 2.0 and 4.0 V are simultaneously achieved. The reaction mechanism behind the superb performance regarding structure evolution and valence variation of Cu/Fe/Mn is monitored by in operando X‐ray diffraction (XRD) and ex situ X‐ray photoelectron spectroscopy (XPS). Moreover, through galvanostatic intermittent titration technique (GITT), cyclic voltammetry (CV), electrochemical impedance spectroscopy (EIS), and density functional theory (DFT) computations, the electrode process kinetics concerning Na‐ion diffusivity and Na^+^ migration energy barrier are systematically investigated. In addition, a soft‐package sodium‐ion full battery employing the as‐prepared P2‐Na_0.76_Cu_0.22_Fe_0.30_Mn_0.48_O_2_ cathode and the hard carbon anode is well established, exhibiting bright practical prospects.

## 2 Results and Discussion

**Figure 1:**
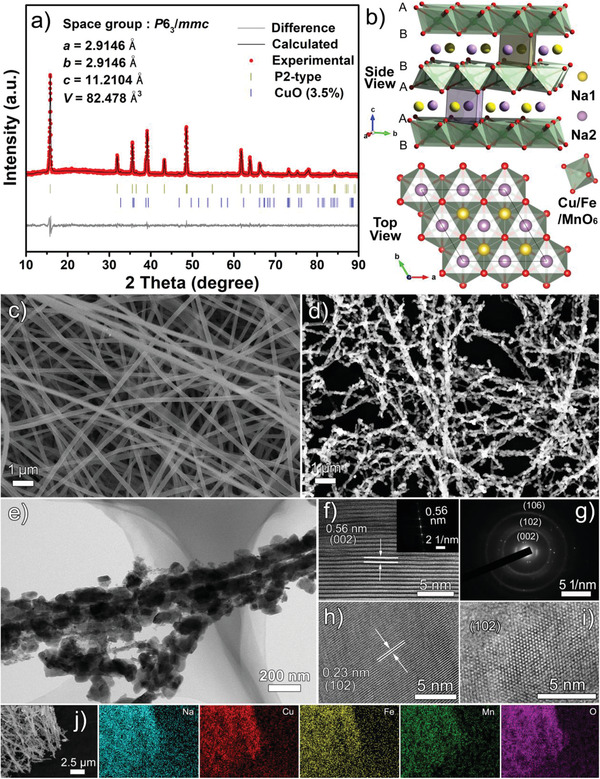
Structural and morphological characterizations: a) Rietveld refined XRD pattern with corresponding cell parameters of the as‐prepared Na_0.76_Cu_0.22_Fe_0.30_Mn_0.48_O_2_. b) Schematic illustration of the P2‐type crystal structure. SEM images of the c) electrospun nanofibers and d) resultant Na_0.76_Cu_0.22_Fe_0.30_Mn_0.48_O_2_ nano‐necklaces. e) TEM image, f) HRTEM image with an inset of FFT image, g) SAED pattern, h,i) HRTEM images, and j) SEM‐EDS mapping images of the Na_0.76_Cu_0.22_Fe_0.30_Mn_0.48_O_2_ nano‐necklaces.

The target P2‐Na_0.76_Cu_0.22_Fe_0.30_Mn_0.48_O_2_ nano‐necklaces were successfully synthesized via a facile electrospinning method followed by annealing processes. As shown in **Figure** **1**a, the XRD Rietveld refinement of the as‐prepared Na_0.76_Cu_0.22_Fe_0.30_Mn_0.48_O_2_ reveals that most of the diffraction peaks can be indexed to a hexagonal P2‐structure with a space group *P*6_3_ */mmc* (JCPDS No. 54–0894), meanwhile, a good agreement between the experimental and the calculated data is obtained (*R*_wp_ = 8.55%). Due to the limited solubility of Cu^2+^ in the P2‐structure, the presence of minor CuO impurity (3.5%) is confirmed. This phenomenon has also been reported before in other copper‐doped P2‐phase materials.^[^ ^40^, ^46^ ^]^ The lattice parameters, which are refined to be *a* = *b* = 2.9146 Å, *c* = 11.2104 Å, and *V* = 82.478 Å^3^, turn out to be bigger than those of the standard P2‐structured material resulting from the larger ionic radius of Cu^2+^ (0.73 Å) than that of Fe^3+^ (0.645 Å). This indicates that the copper ions have been successfully incorporated into the TMO_2_ layers, providing more interstitial spaces for Na^+^ ions diffusion.^[^ ^40^, ^41^ ^]^ The detailed structure information regarding lattice parameters, thermal factors, agreement factors, atomic sites, and occupancies determined by the Rietveld refined XRD is exhibited in Table S1 (Supporting Information). The P2‐type crystalline structure acquired from the fitted results is plotted in Figure 1b. Along the *c*‐axis, oxygen slabs are stacked in an ABBAAB sequence and sodium ions are located at two different prismatic sites, namely, Na1 and Na2 sharing faces and edges with MO_6_ octahedra, respectively.^[^ ^38^ ^]^ The Cu/Fe/Mn ions occupy the octahedral sites of TMO_2_ layers in a random distribution. Figure 1c clearly displays the scanning electron microscopy (SEM) image of the electrospun precursor sample, presenting smooth and continuous nanofibers that weave into a 3D network. After a thermal treatment at 850 °C for 6 h, the continuous 1D nanostructures are inherited and still maintain an average diameter of around 200 nm (Figure 1d); myriads of irregular nanograins string tightly together to constitute the pearl necklace‐like hierarchitectures. When viewed at a lower magnification, the as‐spun nanofibers and the annealed nano‐necklaces both demonstrate a wide‐range and uniform production (Figure S1, Supporting Information). The microstructure is further scrutinized by transmission electron microscopy (TEM) image (Figure 1e) and high‐magnification SEM image (Figure S2 in the Supporting Information), disclosing that the sizes of the secondary nanograins mostly range from 50 to 150 nm. To the best of our knowledge, these are the smallest particles ever reported for the Fe/Mn‐based layered oxides. The high‐resolution TEM (HRTEM) image is subsequently presented in Figure 1f, intuitively corroborating the layered structure of P2‐Na_0.76_Cu_0.22_Fe_0.30_Mn_0.48_O_2_ with the (002) interplanar distance of 0.56 nm, as is also validated in the fast Fourier transform (FFT) image (inset of Figure 1f). The selected‐area electron diffraction (SAED) pattern (Figure 1g) featuring concentric rings suggests the polycrystalline nature of the Na_0.76_Cu_0.22_Fe_0.30_Mn_0.48_O_2_ nanograins. The narrower lattice fringes with an interplanar spacing of 0.23 nm can be well attributed to the (102) plane (Figure 1h). Meanwhile, the high‐angle annular dark field (HAADF) TEM image (Figure 1i) also exhibits the (102) plane and the bright dots depict the positions of transition metal (Cu/Fe/Mn) atoms. A homogenous distribution of elements in the P2‐Na_0.76_Cu_0.22_Fe_0.30_Mn_0.48_O_2_ nano‐necklaces such as Na, Cu, Fe, Mn, and O is verified by energy dispersive spectroscopy (EDS) mapping (Figure 1j). Besides, in order to gain the ideal structure and morphology, the calcination temperature and time have been adjusted reasonably (Figures S3 and S4 in the Supporting Information). It is found that a mixture of P3 + P2 phases is obtained at a low temperature of 750 °C. With the temperature rising to 800 °C, the percentage of P3 phase is decreasing and the P2 phase gradually plays a dominant role. Finally, the pure phase of P2‐Na_0.76_Cu_0.22_Fe_0.30_Mn_0.48_O_2_ is achieved at 850 °C. Moreover, a sintering time of 2 h is too short for the target necklace‐morphology to form uniformly, while a long time (10 h) will cause the collapse of necklace‐framework and the local agglomeration of oxide nanograins. Therefore, it is believed that an annealing temperature of 850 °C with a time of 6 h should be the most appropriate condition in this work.

TEM‐EDS spectrum and mapping images (Figure S5, Supporting Information) reveal that the molar ratio of Na:Cu:Fe:Mn in the as‐prepared oxide is 0.76:0.22:0.30:0.48, and the elements are homogenously distributed in a single nanograin. The inductively coupled plasma (ICP) analysis further ascertains that the Na/Cu/Fe/Mn molar ratio is in good accordance with the anticipated values. The chemical compositions of the Na_0.76_Cu_0.22_Fe_0.30_Mn_0.48_O_2_ nano‐necklaces have also been investigated by XPS, no peaks other than Na, Cu, Fe, Mn, and O elements appear in the survey spectrum (Figure S6, Supporting Information), indicating the high purity of the product. In addition, N_2_ adsorption–desorption measurement was conducted on the Na_0.76_Cu_0.22_Fe_0.30_Mn_0.48_O_2_ sample (Figure S7, Supporting Information), displaying a typical type‐IV behavior with an obvious hysteresis loop at relative pressure (*P*/*P*_0_) ranging from 0.5 to 1.0. Correspondingly, the specific surface area is 66.58 m^2^ g^−1^ calculated from the Brunauere–Emmette–Teller method, and the Barrette–Joynere–Halenda pore sizes are between 2 and 20 nm.

**Figure 2:**
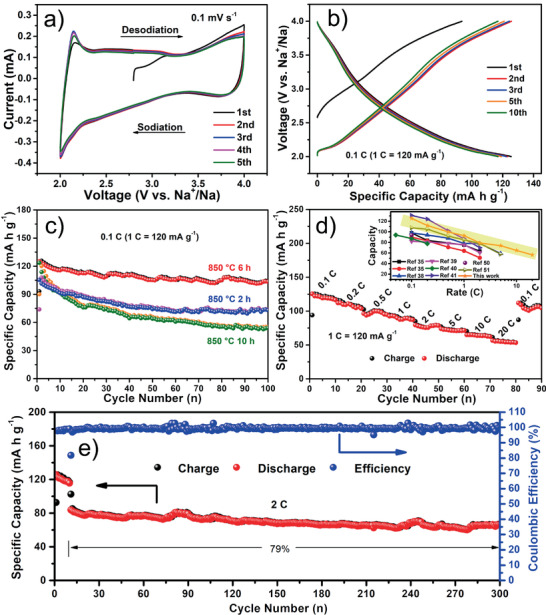
Electrochemical properties: a) CV curves at a scan rate of 0.1 mV s^−1^ and b) galvanostatic charge/discharge profiles at 0.1 C in the voltage window of 2.0–4.0 V (vs Na^+^/Na) of the P2‐Na_0.76_Cu_0.22_Fe_0.30_Mn_0.48_O_2_ nano‐necklaces cathode. c) Cycling performance of the Na_0.76_Cu_0.22_Fe_0.30_Mn_0.48_O_2_ nano‐necklaces (850 °C for 6 h) in comparison with the counterparts annealed at 850 °C for 2 and 10 h at 0.1 C. d) Rate capability of the Na_0.76_Cu_0.22_Fe_0.30_Mn_0.48_O_2_ nano‐necklaces cathode, inset is the comparison between this work and the previously reported Cu‐doped Fe/Mn‐based layered oxides Na‐storage cathode materials. e) Long‐term cycling life of the Na_0.76_Cu_0.22_Fe_0.30_Mn_0.48_O_2_ nano‐necklaces at 2 C.

Prior to electrochemical measurements on the P2‐Na_0.76_Cu_0.22_Fe_0.30_Mn_0.48_O_2_ nano‐necklaces cathode, CV and galvanostatic charge/discharge tests were first performed on the P2‐Na_2/3_Fe_1/2_Mn_1/2_O_2_ sample, which was prepared using the similar method without the addition of Cu source. Figure S8a (Supporting Information) shows the CV curves of the P2‐Na_2/3_Fe_1/2_Mn_1/2_O_2_ cathode cycled at a scan rate of 0.1 mV s^−1^ between 2.0 and 4.0 V. The anodic peaks at potentials of 3.45 and 3.9 V, as well as the cathodic peak at around 3.8 V, correspond to the redox reactions of the Fe^3+^/Fe^4+^ couple;^[^ ^34^, ^38^ ^]^ while the peaks below 2.5 V should be attributed to the redox reactions of the Mn^3+^/Mn^4+^ couple.^[^ ^46^, ^47^ ^]^ Compared with the CV profiles of the Na_0.76_Cu_0.22_Fe_0.30_Mn_0.48_O_2_ nano‐necklaces cathode in **Figure** **2**a, the main difference is that the oxidation peak at 3.45 V related to the abovementioned Fe^3+^/Fe^4+^ redox process disappears and the curves become more sloping and flat. This phenomenon can be interpreted as the Fe^3+^/Fe^4+^ redox peaks are able to merge with the oxidation/reduction peaks of Cu^2+^/Cu^3+^ (4.0/3.9 V).^[^ ^38^, ^40^ ^]^ Similar results are also observed in the charge/discharge profiles of the two electrodes. As elucidated in Figure S8b (Supporting Information), several voltage plateaus of the P2‐Na_2/3_Fe_1/2_Mn_1/2_O_2_ electrode are in great coincidence with the CV study and there is a distinct plateau located at ≈3.45 V on the charge curve, which is related to the Na^+^/vacancy ordering due to the gliding of oxygen planes, resulting in the formation of some unfavorable intermediate phases.^[^ ^34^, ^48^ ^]^ Note that although the P2‐Na_2/3_Fe_1/2_Mn_1/2_O_2_ electrode delivers a high initial discharge capacity of 129.9 mA h g^−1^, it suffers from fast capacity decay with a capacity retention of only 35.5% after 100 cycles (Figure S8c, Supporting Information). Figure 2b plots the charge/discharge profiles of the Na_0.76_Cu_0.22_Fe_0.30_Mn_0.48_O_2_ nano‐necklaces at a current density of 0.1 C (1 C = 120 mA g^−1^), the electrode delivers an initial discharge capacity of 125.5 mA h g^−1^, corresponding to ≈0.50 e^−^ transfer per formula unit. The profiles become smoother and possess better overlapping and smaller voltage hysteresis compared to those of the Na_2/3_Fe_1/2_Mn_1/2_O_2_ sample, indicating an improved reversibility and enhanced reaction kinetics. More importantly, no obvious voltage plateau is found at around 3.45 V on the charge curves, suggesting that the Cu substitution of Fe can efficaciously mitigate the Na^+^/vacancy ordering. It is believed that Cu^2+^ can serve as a stabilizer blocking the rearrangement of Na^+^/vacancy, alleviating the Jahn–Teller effect of Mn^3+^ center upon sodiation/desodiation (diluting the Mn^3+^ concentration), and suppressing the irreversible Fe migration into the Na sites upon charging to high voltage.^[^ ^35^, ^38^, ^40^, ^41^, ^49^ ^]^ Hence, the structural stability is significantly improved through an approximate solid‐solution process during cycling. This point is further evidenced by the HRTEM images of the Na_2/3_Fe_1/2_Mn_1/2_O_2_ and Na_0.76_Cu_0.22_Fe_0.30_Mn_0.48_O_2_ electrode materials after ten charge/discharge cycles (Figure S9 in the Supporting Information). The un‐doped sample shows obvious lattice distortions, while the layered structure of the Cu‐doped sample is well reserved. Figure 2c illustrates the cycling performance of the Na_0.76_Cu_0.22_Fe_0.30_Mn_0.48_O_2_ samples thermally treated at 850 °C for 2, 6, and 10 h, respectively. Benefiting from the special morphology, the Na_0.76_Cu_0.22_Fe_0.30_Mn_0.48_O_2_ nano‐necklaces cathode could deliver a reversible capacity of 103.6 mA h g^−1^ at 0.1 C after 100 cycles with a promising capacity retention of 82.7%. Post‐mortem examination in SEM image (Figure S10a, Supporting Information) shows a well‐maintained architecture of the P2‐Na_0.76_Cu_0.22_Fe_0.30_Mn_0.48_O_2_ nano‐necklaces after cycling. The not well‐formed hierarchical nanostructures exposing less active sites lead to an obviously lower capacity for the sample calcinated for 2 h, yet the sample calcinated for 10 h displays a disappointing cyclability with a capacity retention of only 43.8% after 100 cycles, mainly caused by the serious agglomeration of the not well‐aligned nanograins upon repeated sodiation/desodiation (Figure S10b in the Supporting Information). The electrochemical performance of the bulk Na_0.76_Cu_0.22_Fe_0.30_Mn_0.48_O_2_ sample prepared by a sol–gel method (SEM image in Figure S11a, Supporting Information) is also given, exhibiting inferior reversible capacity and cycling performance compared to those of the nano‐necklaces electrode (Figure S11b, Supporting Information). In addition, the content of the doped Cu has been optimized by evaluating the sodium‐storage behaviors of the P2‐type cathode materials with different stoichiometries. As presented in Figure S12 (Supporting Information), the contrast P2‐material containing less Cu shows an inferior cycling stability (≈74.1% capacity retention after 100 cycles) due to the poor inhibiting effects on the Na^+^/vacancy orderings and the Jahn–Teller distortions; while the sample containing excess Cu delivers a relatively low initial discharge capacity of 120.1 mA h g^−1^ at 0.1 C caused by the decreased content of the active Fe^3+^/Fe^4+^ redox couple. These results definitely validate that the present dual‐strategy of Cu doping and hierarchical nanoengineering plays crucial roles in improving the cycling performance of the layered Na‐Fe‐Mn‐O system.

The rate capability of the P2‐Na_0.76_Cu_0.22_Fe_0.30_Mn_0.48_O_2_ nano‐necklaces cathode is presented in Figure 2d. The electrode can release reversible specific capacities of 125.4, 112.0, 96.2, 92.5, 79.5, 73.9, 64.9, and 56.5 mA h g^−1^ at 0.1 C, 0.2 C, 0.5 C, 1 C, 2 C, 5 C, 10 C, and 20 C, respectively. More impressively, when the rate is returned to 0.1 C, a discharge capacity of 112.1 mA h g^−1^ can be obtained, attesting the exceptional reversibility during rapid Na^+^ insertion/extraction. The outstanding rate performance of P2‐Na_0.76_Cu_0.22_Fe_0.30_Mn_0.48_O_2_ nano‐necklaces compared with other reported Cu‐doped Fe/Mn‐based layered oxides SIB cathodes is highlighted in the inset of Figure 2d.^[^ ^35^, ^38^, ^39^, ^40^, ^41^, ^50^, ^51^ ^]^ For one reason, the excellent rate capability primarily benefits from the pearl necklace‐like hierarchical nanostructures with high porosity, which shorten the Na^+^ diffusion lengths and provide more open channels for the ionic transportation. The other is that the Cu substitution of Fe brings about an expansion of the interstitial spaces through which sodium ions can facilely diffuse. Last but not least, the copper doping efficiently alleviates the Na^+^/vacancy ordering and thus yields an approximate solid‐solution reaction mechanism, rendering the fast Na‐ion diffusivity. Thereafter, the long‐term cycling performance at 2 C is displayed in Figure 2e, where the P2‐Na_0.76_Cu_0.22_Fe_0.30_Mn_0.48_O_2_ nano‐necklaces electrode enables a respectable capacity retention of 79% after 300 cycles, reaching the highest value for the Cu‐doped Fe/Mn‐based layered oxides SIB cathodes till now (Table S2, Supporting Information).

**Figure 3:**
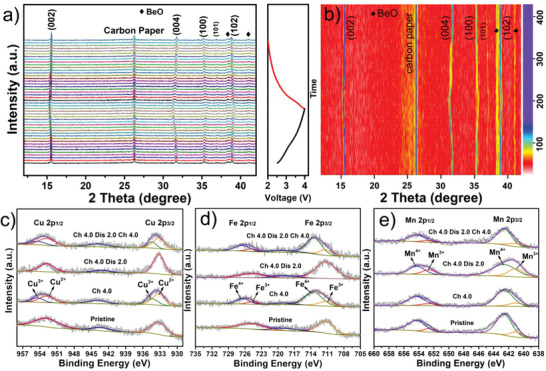
Na‐storage mechanism: a) in situ XRD patterns of the P2‐Na_0.76_Cu_0.22_Fe_0.30_Mn_0.48_O_2_ nano‐necklaces electrode during the first charge/discharge process at 0.1 C, as well as b) the contour plots. Ex situ XPS of high‐resolution c) Cu 2p, d) Fe 2p, and e) Mn 2p spectra at various charged/discharged states of the P2‐Na_0.76_Cu_0.22_Fe_0.30_Mn_0.48_O_2_ cathode.

In order to gain insight into the structure evolution of the Na_0.76_Cu_0.22_Fe_0.30_Mn_0.48_O_2_ nano‐necklaces electrode during the sodium‐ion insertion/extraction process, in operando XRD was conducted within 2.0–4.0 V employing beryllium foil as the testing window and carbon paper as the current collector. The XRD patterns in **Figure** **3**a,b demonstrate a good reversibility in characteristic peaks and the P2‐type structure is well maintained over the entire charge and discharge processes. To be more specific, the (002) and (004) peaks gradually shift leftward upon charging from 2.0 to 4.0 V, while the (100) and (102) peaks shift rightward at the meantime. These phenomena can be interpreted as the extraction of Na^+^ gives rise to an increased repulsive electrostatic interaction of O^2−^ from the neighboring transition metal layers, thus leading to an expansion of the interlayer spacing and a contraction of the TMO_6_ octahedra, which reflect on the increase of *c* parameter and the decrease of *a* parameter. Upon discharging from 4.0 to 2.0 V, an eminent reversibility can be observed, since all the abovementioned peaks move toward the opposite direction compared to the shift in charging process. It is found that the (00*l*) peaks move to higher angles and the (10*l*) peaks move to lower angles at the end of discharging, compared with the pristine state. This is resulted from the extra high Coulombic efficiency in the initial cycle (as shown in Figure 2b). Moreover, it is worth noticing that the peaks of (004) and (100) split into two peaks in the high‐voltage region but swiftly merge into one peak along with the voltage decreasing, indicating the transient existence of a two‐phase reaction region caused by the Na^+^/vacancy ordering, meanwhile, proving the good reversibility of the Cu‐doped samples. The lattice parameters evolution of P2‐Na_0.76_Cu_0.22_Fe_0.30_Mn_0.48_O_2_ upon charge/discharge through Rietveld refinements on the in situ XRD patterns is added in Figure S13 (Supporting Information), revealing a volume change of only 1.43% from the pristine to the fully charged states, much smaller than that of P2‐Na_2/3_Fe_1/2_Mn_1/2_O_2_ (≈7.7%).^[^ ^52^ ^]^ This indicates that Cu‐doping can effectively alleviate the volume change and stabilize the structure during electrochemical cycling. Besides, the surface oxidation states of the P2‐Na_0.76_Cu_0.22_Fe_0.30_Mn_0.48_O_2_ nano‐necklaces electrode during cycling were investigated by ex situ XPS. Figure 3c,d displays the core‐level XPS spectra of Cu 2p and Fe 2p at different charged/discharged states: the pristine electrode featuring Cu 2p_1/2_ (952.7 eV), Cu 2p_3/2_ (933.2 eV), and a satellite peak (943.3 eV) implies the presence of Cu^2+^;^[^ ^38^, ^49^ ^]^ and the Fe 2p_1/2_ and Fe 2p_3/2_ peaks (accompanied by a satellite peak) located at about 724.6 and 711.3 eV, respectively, indicate the existence of Fe^3+^.^[^ ^38^, ^41^ ^]^ When fully charged to 4.0 V, the peaks of Cu 2p and Fe 2p both move to higher binding energy; and the deconvoluted results manifest the transitions of a small amount of Cu^2+^ to Cu^3+^ and a considerable amount of Fe^3+^ to Fe^4+^ upon Na^+^ extraction. When fully discharged to 2.0 V, all of the peaks recover to the pristine state, verifying that the Cu^2+^/Cu^3+^ and Fe^3+^/Fe^4+^ redox couples are both active and experience highly reversible change in the potential range of 2.0–4.0 V. Afterward, the Na_0.76_Cu_0.22_Fe_0.30_Mn_0.48_O_2_ electrode was re‐charged to 4.0 V, the valence transitions of Cu^2+^ to Cu^3+^ and Fe^3+^ to Fe^4+^ could be observed again. As for the Mn 2p core‐level spectra (Figure 3d): the deconvoluted results of Mn 2p_1/2_ and Mn 2p_3/2_ peaks indicate the coexistence of Mn^3+^ and Mn^4+^ in the pristine electrode.^[^ ^41^, ^47^ ^]^ Upon charging to 4.0 V, no obvious change can be detected; when discharged to 2.0 V, a small amount of the Mn^4+^ is reduced to Mn^3+^; when charged to 4.0 V again, the Mn^3+^ is re‐oxidized to Mn^4+^. These findings prove that the Cu^2+/3+^, Fe^3+/4+^, and Mn^4+/3+^ redox couples all take part in the electrochemical reaction and collaboratively contribute to the reversible capacity of the P2‐Na_0.76_Cu_0.22_Fe_0.30_Mn_0.48_O_2_ cathode within 2.0–4.0 V. In addition, the reversible sodiation and desodiation processes of P2‐Na_0.76_Cu_0.22_Fe_0.30_Mn_0.48_O_2_ can be further tracked by ex situ Raman spectra (Figure S14, Supporting Information), which show that the characteristic peaks involving Na vibrations conspicuously weaken during charging, then significantly strengthen again upon discharging.

**Figure 4:**
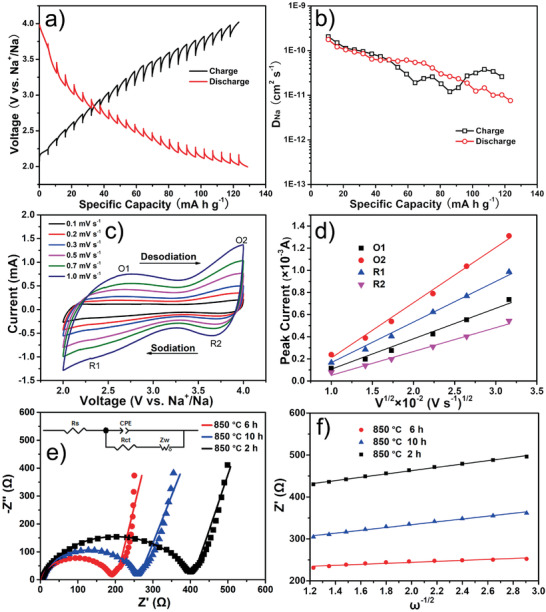
Electrode process kinetics: a) charge–discharge GITT profiles of the P2‐Na_0.76_Cu_0.22_Fe_0.30_Mn_0.48_O_2_ nano‐necklaces cathode at 15 mA g^−1^ and b) the corresponding Na^+^ diffusion coefficients (*D*_Na_). c) CV curves at different scan rates from 0.1 to 1.0 mV s^−1^ and d) the corresponding fitting curves between peak currents (*i*_p_) and square root of scan rates (*v* ^1/2^). e) Nyquist dots and fitting curves of the samples annealed at 850 °C for 2, 6 (P2‐Na_0.76_Cu_0.22_Fe_0.30_Mn_0.48_O_2_ nano‐necklaces), and 10 h, as well as f) the corresponding real parts of the impedance (*Z*′) versus reciprocal square root of the angular frequency (*ω*) in the low frequency region, inset is the equivalent circuit.

For a better understanding of the origin of the outstanding rate performance of P2‐Na_0.76_Cu_0.22_Fe_0.30_Mn_0.48_O_2_ nano‐necklaces, the electrode process kinetics were systematically evaluated by means of GITT measurements, CV curves at various sweep rates, and EIS spectra. The typical charge/discharge GITT profile in the second cycle is displayed in **Figure** **4**a, and the time versus voltage profile for a single titration is plotted in Figure S15 (Supporting Information), showing that the cell experiences an uninterrupted current flux (15 mA g^−1^) for 20 min during charging or discharging and then a relaxing time for 80 min to reach an equilibrium of voltage. Figure 4b exhibits the apparent Na‐diffusion coefficients (*D*_Na_) as a function of specific capacity. The *D*_Na_ values of P2‐Na_0.76_Cu_0.22_Fe_0.30_Mn_0.48_O_2_ (10^−10^ to 10^−11^ cm^2^ s^−1^) are higher than those of the P2‐Na_2/3_Fe_1/2_Mn_1/2_O_2_ cathode (10^−11^ to 10^−12^ cm^2^ s^−1^ acquired from Figure S16, Supporting Information). This result indicates that the existence of multi‐phase reaction caused by the Na^+^/vacancy ordering in P2‐Na_2/3_Fe_1/2_Mn_1/2_O_2_ hinders the sodium‐ion mobility, whereas the above issue can be well relieved by the substitution of Fe by Cu. Figure 4c shows the representative CV curves of the P2‐Na_0.76_Cu_0.22_Fe_0.30_Mn_0.48_O_2_ electrode at different sweep rates from 0.1 to 1.0 mV s^−1^. The CV curves maintain similar shapes and exhibit a slight polarization as the sweep rate increases, reflecting the decent reaction kinetics upon Na^+^ (de)insertion. The *D*_Na_ can be calculated based on the CV study according to the Randles–Sevcik formula, i.e., *i*_p_ = 2.69 × 10^5^ *n* ^3/2^ *AD*_Na_^1/2^ *C*_Na_ *v* ^1/2^, where *i*_p_ (A) is the peak current, *v* (V s^−1^) is the scan rate, *C*_Na_ is the Na ions concentration in the electrode (≈3.06 × 10^−2^ mol cm^−3^), *A* (cm^2^) is the contacting area (0.81 cm^2^) between electrode and electrolyte, and *n* is the transferred electron number per molecule (*n* = 0.50 in this system).^[^ ^20^, ^47^ ^]^ The *i*_p_ of the four main redox peaks displays a good linear relationship with the square root of *v* in Figure 4d, accordingly, the *D*_Na_ values for the O1, O2, R1, and R2 peaks are 1.48 × 10^−10^, 4.43 × 10^−10^, 2.59 × 10^−10^, and 9.11 × 10^−11^ cm^2^ s^−1^, respectively, coinciding well with the GITT results. The CV profiles at different scan rates also reveal the pseudo‐capacitance contributions to the charge storage process, rendering the rapid reaction kinetics of the P2‐Na_0.76_Cu_0.22_Fe_0.30_Mn_0.48_O_2_ nano‐necklaces cathode. The capacitive effect gradually strengthens with the increase of scan rates (Figure S17 in the Supporting Information). Furthermore, EIS spectra were employed to estimate the *D*_Na_ in the three samples with different annealing conditions. The Nyquist plots in Figure 4e consist of a semicircle from high to medium frequency corresponding to the charge‐transfer resistance (*R*_ct_) at the electrode/electrolyte interface, and a slope line at low frequency correlated to the Warburg impedance (*Z*_w_) associated with Na^+^ diffusion. The *D*_Na_ values can be determined from the linear fitting results in Figure 4f (calculation details are provided in the Supporting Information). The P2‐Na_0.76_Cu_0.22_Fe_0.30_Mn_0.48_O_2_ nano‐necklaces electrode (850 °C for 6 h) possesses the lowest resistance of about 180 Ω and the fastest Na^+^ diffusivity of around 10^−11^ cm^2^ s^−1^, which can be attributed to its favorable pearl necklace‐like hierarchical nanostructures constituted by small secondary nanograins that offer abundant rapid paths for electronic/ionic transport. In addition, the resistance of P2‐Na_0.76_Cu_0.22_Fe_0.30_Mn_0.48_O_2_ is smaller than that of P2‐Na_2/3_Fe_1/2_Mn_1/2_O_2_ (about 290 Ω acquired from Figure S18, Supporting Information), unambiguously confirming the positive effect of Cu‐doping (creating charge defects) on enhancing the electronic conductivity.

**Figure 5:**
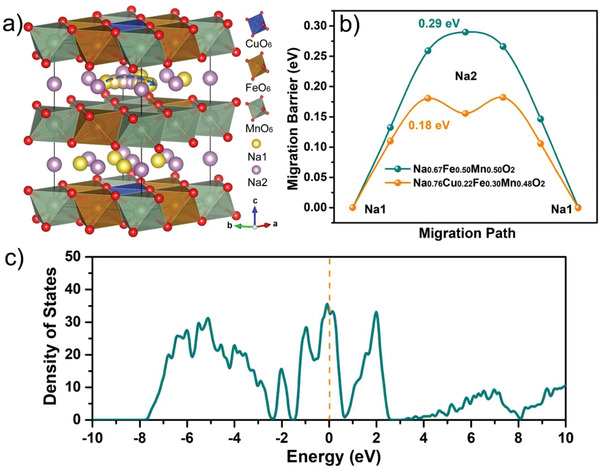
DFT computations: a) schematic crystal structure of P2‐Na_0.76_Cu_0.22_Fe_0.30_Mn_0.48_O_2_ displaying the optimum Na‐ion migration pathway (depicted by the blue arrow), and b) the corresponding migration energy barriers following the Na1→Na2→Na1 route in the Na_2/3_Fe_1/2_Mn_1/2_O_2_ and Na_0.76_Cu_0.22_Fe_0.30_Mn_0.48_O_2_ lattices. c) DOS of the P2‐Na_0.76_Cu_0.22_Fe_0.30_Mn_0.48_O_2_.

To figure out the root of the excellent electrochemical performance of the P2‐Na_0.76_Cu_0.22_Fe_0.30_Mn_0.48_O_2_ electrode in depth, the Na‐ion migration paths and energy barriers were explored via first‐principles computations based on the DFT. **Figure** **5**a shows the primitive cell of P2‐Na_0.76_Cu_0.22_Fe_0.30_Mn_0.48_O_2_, where the blue arrow points out the optimum migration path following Na1→Na2→Na1. Note that the atomic positions of Na, Cu, Fe, and Mn are simulated based on their atomic ratios and occupancies (Table S1 in the Supporting Information), and the migration path near the Cu‐doping site is selected to figure out the effect of Cu‐doping on the Na^+^ migration behavior in the layered oxide lattice. As elucidated in Figure 5b, the Na^+^ migration barrier in the Na_0.76_Cu_0.22_Fe_0.30_Mn_0.48_O_2_ crystal is calculated to be about 0.18 eV using the climbing image nudged elastic band (cNEB) method, while the ionic migration barrier in Na_2/3_Fe_1/2_Mn_1/2_O_2_ is 0.29 eV. This result provides another solid evidence that the substitution of Fe by Cu is beneficial for the facile Na^+^ diffusion. It is noteworthy that the sodium‐ion diffusion barrier in Na_0.76_Cu_0.22_Fe_0.30_Mn_0.48_O_2_ is much lower than those in other reported Na‐storage cathode materials (mostly ≥0.3 eV),^[^ ^53^ ^]^ accounting for the superb reaction kinetics of the present material. Interestingly, the projected density of states (DOS) of P2‐Na_0.76_Cu_0.22_Fe_0.30_Mn_0.48_O_2_ manifest some metallic properties (Figure 5c), which are responsible for the distinguished rate capability due to the desirable electronic conductivity. Meanwhile, the DOS of Na_2/3_Fe_1/2_Mn_1/2_O_2_ is presented in Figure S19 (Supporting Information).

**Figure 6:**
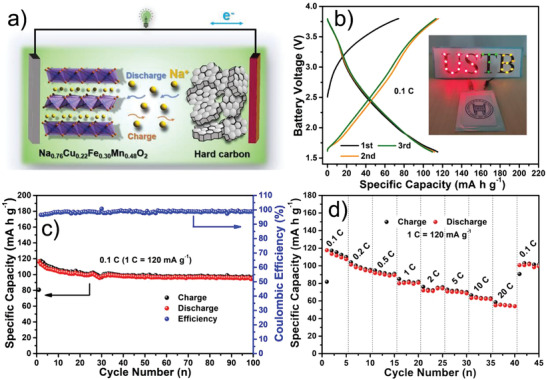
Sodium‐ion full batteries: a) diagram of the P2‐Na_0.76_Cu_0.22_Fe_0.30_Mn_0.48_O_2_ cathode//hard carbon anode full battery. b) Galvanostatic charge/discharge profiles and c) cycling performance at 0.1 C in the voltage window of 1.6–3.8 V of the full battery (based on the cathode active mass), inset is the digital photo showing LED bulbs powered by the pouch cell. d) Rate performance of the full battery.

The appealing half‐cell performance of the P2‐Na_0.76_Cu_0.22_Fe_0.30_Mn_0.48_O_2_ cathode motivates us to further examine its full‐cell properties, focusing on preliminarily assessing the practicability in a Na‐based battery system. Herein, a prototype full cell configuration schematized in **Figure** **6**a was designed by coupling the P2‐Na_0.76_Cu_0.22_Fe_0.30_Mn_0.48_O_2_ nano‐necklaces cathode with the hard carbon anode. The hard carbon anode can deliver a reversible capacity of around 210 mA h g^−1^ with an operating potential of ≈0.13 V versus Na^+^/Na (Figure S20, Supporting Information). In this case, the active mass ratio of cathode to anode was set at 1.7:1 to balance the capacity. The full cell was tested at 0.1 C within the voltage window of 1.6–3.8 V, a reversible discharge capacity of 115 mA h g^−1^ along with an average working voltage of ≈2.45 V (based on the cathode active mass) can be acquired (Figure 6b), enabling a remarkable energy density of 177.4 Wh kg^−1^ based on the total mass of cathode and anode active materials. Besides, light‐emitting diodes (LEDs) with different colors could be successfully lightened up by the pouch cell (inset of Figure 6b). Figure 6c shows that a discharge capacity of 95.4 mA h g^−1^ is maintained after 100 cycles, corresponding to a promising capacity retention of 82%. The rate profiles depicted in Figure 6d further reveal that prominent discharge capacities of 117.7 mA h g^−1^ at 0.1 C and 55 mA h g^−1^ at 20 C can be obtained.

## 3 Conclusion

To sum up, a dual‐strategy of cation‐doping and nanoengineering was applied to obtain the pearl necklace‐like hierarchical nanostructures of a novel P2‐Na_0.76_Cu_0.22_Fe_0.30_Mn_0.48_O_2_ cathode through a feasible electrospinning method. On the one hand, the unique 1D nanostructures assembled by small nanograins could facilitate the accessibility to electrolyte, offer facile pathways for electrons/Na^+^ ions transportation, and maintain the structural integrity upon repetitive Na^+^ insertion/extraction. On the other hand, the Cu substitution of Fe created larger interstitial spaces in the oxide crystal through which sodium ions could rapidly diffuse, and efficaciously mitigated the Na^+^/vacancy ordering during cycling. Thus, an exceptional rate capability (125.4 mA h g^−1^ at 0.1 C in comparison with 56.5 mA h g^−1^ at 20 C) and a fascinating cyclability (≈79% capacity retention after 300 cycles) were simultaneously attained. In operando XRD and ex situ XPS were employed to track the highly reversible structure evolution and Cu/Fe/Mn valence change upon charge/discharge, and the positive effects of Cu doping on the reaction kinetics were further confirmed by GITT, CV, EIS, and DFT computations. More intriguingly, the prototype Na‐ion full battery constructed by the P2‐Na_0.76_Cu_0.22_Fe_0.30_Mn_0.48_O_2_ nano‐necklaces cathode and hard carbon anode delivered a distinguished energy density of 177.4 Wh kg^−1^, demonstrating a great promise for practical applications. We believe that the combination of metallic cation doping with hierarchical nanoengineering provides a new and ideal platform for enhancing the electrochemical performance of the layered oxide cathode materials for SIBs.

## 4 Experimental Section

##### Materials Synthesis

The Na_0.76_Cu_0.22_Fe_0.30_Mn_0.48_O_2_ nano‐necklaces were fabricated via a feasible electrospinning method with subsequent heat treatments. In order to prepare the precursor solution, 4.4 g of polyvinylpyrrolidone (average Mw = 13 00 000, Aladdin) was first dissolved in 30 mL of deionized water under magnetic stirring at room temperature. After complete dissolution, 4 mL of CH_3_COOH (99.5%, Aladdin) was added to create an acidic environment. Then, 3.6 mmol of Mn(CH_3_COO)_2_·4H_2_O (99.9%, Aldrich), 5.985 mmol (5% excess) of NaNO_3_ (99.9%, Aladdin), 1.65 mmol of Cu(NO_3_)_2_·3H_2_O (99.9%, Aladdin), and 2.25 mmol of Fe(NO_3_)_3_·9H_2_O (99.9%, Aladdin) were successively put into the above solution with fully mixing.

After overnight stirring, electrospinning was carried out by loading the as‐prepared fluid into a plastic syringe equipped with a 21‐gauge blunt‐tip needle. The solution was electrospun by a high voltage of 17 kV applied between the needle tip and the aluminium foil collector, with a distance of 15 cm, at a feeding rate of 15 µL min^−1^. Consequently, an ≈150 µm thick membrane could be obtained by electrospinning at 35 °C for 36 h.

After electrospinning, the as‐spun membrane was peeled off from the Al foil collector and thermally treated in a following step‐wise process: 350 °C for 2 h (heating rate: 1 °C min^−1^), 500 °C for 2 h (heating rate: 2 °C min^−1^), and 850 °C for 6 h (heating rate: 3 °C min^−1^) in ambient air. Adopting slow heating rates in a step‐wise calcination process was to protect the pearl necklace‐like nanostructure from collapse. After careful calculation, the cost for synthesizing 100 mg of the target nano‐necklace materials was around 4.59 USD. This implied that the present nanoengineering technique was relatively cost‐effective, and was suitable for large‐scale production by adopting the multi‐nozzle equipment. Besides, the final calcination temperature (750, 800, or 850 °C) and time (2, 6, or 10 h) were modulated to optimize the synthesis conditions. In order to figure out the effects of Cu substitution, P2‐Na_2/3_Fe_1/2_Mn_1/2_O_2_, P2‐Na_0.76_Cu_0.20_Fe_0.32_Mn_0.48_O_2_, and P2‐Na_0.76_Cu_0.24_Fe_0.28_Mn_0.48_O_2_ samples were also prepared through electrospinning and annealing procedures under the similar conditions to the abovementioned P2‐Na_0.76_Cu_0.22_Fe_0.30_Mn_0.48_O_2_ sample used, except for changing the metal salts dosage ratio according to the stoichiometry. The bulk Na_0.76_Cu_0.22_Fe_0.30_Mn_0.48_O_2_ was synthesized by a sol–gel method using the same metal salts dosage as that in the preparation of the nano‐necklaces sample, with extra addition of the complexing agent (citric acid). The annealing processes included 400 °C for 4 h and then 850 °C for 6 h in air (heating rate: 5 °C min^−1^).

##### Materials Characterizations

Crystallographic properties of the annealed products were determined by XRD (Rigaku D/Max‐2500) with filtered Cu K*α* radiation (*λ* = 1.5418 Å) in the 2*θ* range of 10°–90°. Morphological and microstructural studies were scrutinized by field‐emission scanning electron microscopy (FE‐SEM, JEOL JSM7500F) and TEM (TECNAI G2 20). Elemental analyses were ascertained by SEM‐ and TEM‐energy dispersive X‐ray spectrometer (EDS) mapping. Nitrogen adsorption–desorption isotherms obtained on a Micromeritics ASAP 2460 instrument (MicroActive) at 77 K were applied to evaluate the porosity and specific surface area. Raman spectra were gathered on a confocal Raman microscope (Thermo‐Fisher Scientific) using an argon‐ion laser (*λ* = 532 nm) in ambient air. The chemical content was ascertained by ICP atomic emission spectroscopy (ICP‐AES, Varian 725‐ES). X‐ray photoelectron spectrometry (XPS, Perkin Elmer PHI 1600 ESCA) was applied to investigate the valence states of the elements during cycling.

When it comes to in operando XRD characterizations, a particular mold battery was assembled employing beryllium foil as the testing window to allow X‐ray passage and carbon paper as the current collector, thus monitoring in situ reaction during cycling. Each scan was recorded in 0.02° incremental steps between 2*θ* = 12° and 42°. The charge/discharge test was conducted at 0.1 C.

##### Electrochemical Measurements

All electrochemical properties were examined in CR2032 coin‐type cells assembled in a glove box (Mikrouna Universal 2440/750) filled with high‐purity argon. Electrodes were fabricated by casting slurries, the composition of which was 75 wt% active materials, 15 wt% acetylene black, and 10 wt% PVdF dissolved in *N*‐methyl‐2‐pyrrolidone, onto Al foil, followed by drying at 80 °C in vacuum overnight. Then the electrodes were cut into suitable‐size slices with a mass loading of about 2.5 mg cm^−2^. The counter and reference electrode of sodium metal disk was put face‐to‐face with the working electrode sandwiched by a glass fiber separator in the coin cells. 1 m NaClO_4_ in propylene carbonate solution with the addition of 5 vol% fluoroethylene carbonate was employed as the electrolyte. Besides, the assembled cells were placed for 6 h before tests to make sure that the electrolyte soaked into the separators and electrodes completely. The coin cells were galvanostatically charged/discharged on a LAND battery‐test instrument (CT2001A) in a voltage interval of 2.0–4.0 V (vs Na/Na^+^). The tests of cyclic voltammetry within the same voltage region at various scan rates and EIS with the ac perturbation signal of 5.0 mV over the frequency from 100 kHz to 100 mHz were used to study the electrode process kinetics, which were both conducted using a CHI660C electrochemical workstation (Shanghai Chenhua, China). The EIS spectra were recorded at open circuit voltage. Prior to the post‐mortem examinations of the cycled active materials using XRD, XPS, or SEM, the electrodes were taken out from the cells and washed with dimethyl carbonate repeatedly in an argon‐filled glove box.

To construct the full cells, the pre‐sodiated hard carbon was adopted as the anode and well matched with the P2‐Na_0.76_Cu_0.22_Fe_0.30_Mn_0.48_O_2_ cathode. The active mass loading ratio of cathode to anode was set at 1.7:1 to balance the capacity. Finally, the full cell was measured within a voltage window of 1.6–3.8 V.

##### Computational Details

The ionic/electronic conductivity of P2‐Na_0.76_Cu_0.22_Fe_0.30_Mn_0.48_O_2_ and P2‐Na_2/3_Fe_1/2_Mn_1/2_O_2_ was evaluated by ab initio computations based on the DFT via Vienna ab‐initio simulation package (VASP). The projector augmented wave method with the Perdew–Burke–Ernzerhof function was employed. The cut‐off energy and *k*‐point separation were set to 500 eV and 0.03 Å^−1^, respectively. Moreover, the Na‐ion migration paths and barriers were thoroughly ascertained by the bond valence energy landscape and cNEB methods.

## Conflict of Interest

The authors declare no conflict of interest.

## Supporting information

Supporting Information
