# Supplementary material for: Dual‐Strategy of Cation‐Doping and Nanoengineering Enables Fast and Stable Sodium‐Ion Storage in a Novel Fe/Mn‐Based Layered Oxide Cathode
Source: Adv Sci (Weinh). 2020 Sep 24;7(21):2002199. doi: 10.1002/advs.202002199 (PMC7610329; doi:10.1002/advs.202002199)
Supplement: Supplementary file 1 — Supporting Information [file ADVS-7-2002199-s001.pdf]

© 2020 Wiley-VCH GmbH

ADVANCED SCIENCE
Open Access

Supporting Information

for *Adv. Sci.,* DOI: 10.1002/advs.202002199

Dual-Strategy of Cation-Doping and Nanoengineering Enables Fast and Stable Sodium-Ion Storage in a Novel Fe/Mn-Based Layered Oxide Cathode

Qiuyu Shen, Xudong Zhao, Yongchang Liu, Youpeng Li, Jian Zhang, Ning Zhang,* Chenghao Yang, and Jun Chen*

WILEY-VCH

Supporting Information

Dual-Strategy of Cation-Doping and Nanoengineering Enables Fast and Stable Sodium-Ion Storage in a Novel Fe/Mn-Based Layered Oxide Cathode

Qiuyu Shen, Xudong Zhao, Yongchang Liu, Youpeng Li, Jian Zhang, Ning Zhang,* Chenghao Yang, and Jun Chen*

Dr. Q. Shen, Dr. X. Zhao, Prof. Y. Liu
Beijing Advanced Innovation Center for Materials Genome Engineering
Institute for Advanced Materials and Technology
State Key Laboratory for Advanced Metals and Materials
University of Science and Technology Beijing, Beijing 100083, China
E-mail: liuyc@ustb.edu.cn

Prof. N. Zhang
College of Chemistry & Environmental Science
Hebei University, Baoding 071002, China
E-mail: ningzhang@hbu.edu.cn

Dr. Y. Li, Prof. C. Yang
New Energy Research Institute, School of Environment and Energy
South China University of Technology, Guangzhou 510006, China

Dr. J. Zhang, Prof. J. Chen
Beijing Advanced Innovation Center for Materials Genome Engineering
School of Mathematics and Physics
University of Science and Technology Beijing, Beijing 100083, China

Prof. Y. Liu, Prof. N. Zhang
Key Laboratory of Advanced Energy Materials Chemistry (Ministry of Education)
Nankai University, Tianjin 300071, China

Supplementary Figures and Tables

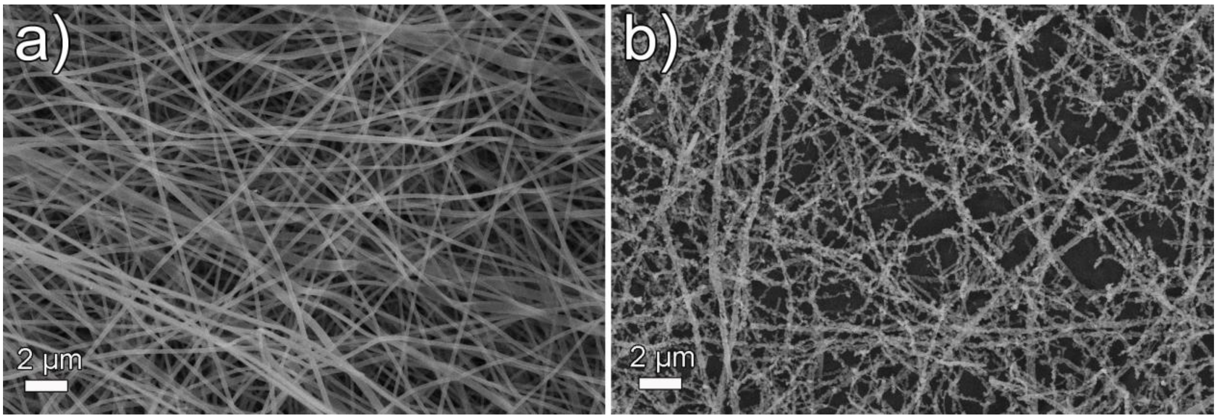

Figure S1. Low-magnification SEM images of the a) electrospun nanofibers and b) annealed P2-$Na_{0.76}Cu_{0.22}Fe_{0.30}Mn_{0.48}O_2$ nano-necklaces.

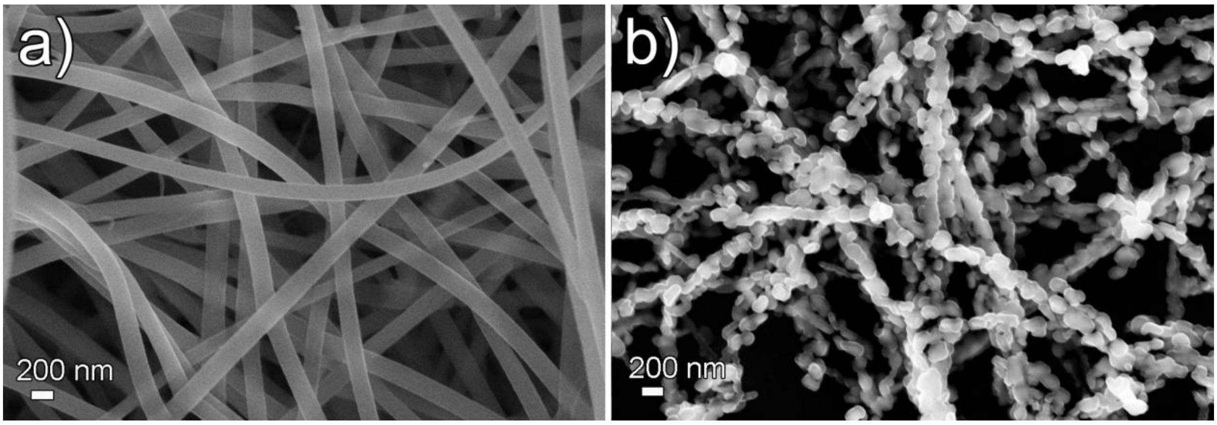

Figure S2. High-magnification SEM images of the a) as-spun nanofibers and b) annealed P2-$Na_{0.76}Cu_{0.22}Fe_{0.30}Mn_{0.48}O_2$ nano-necklaces.

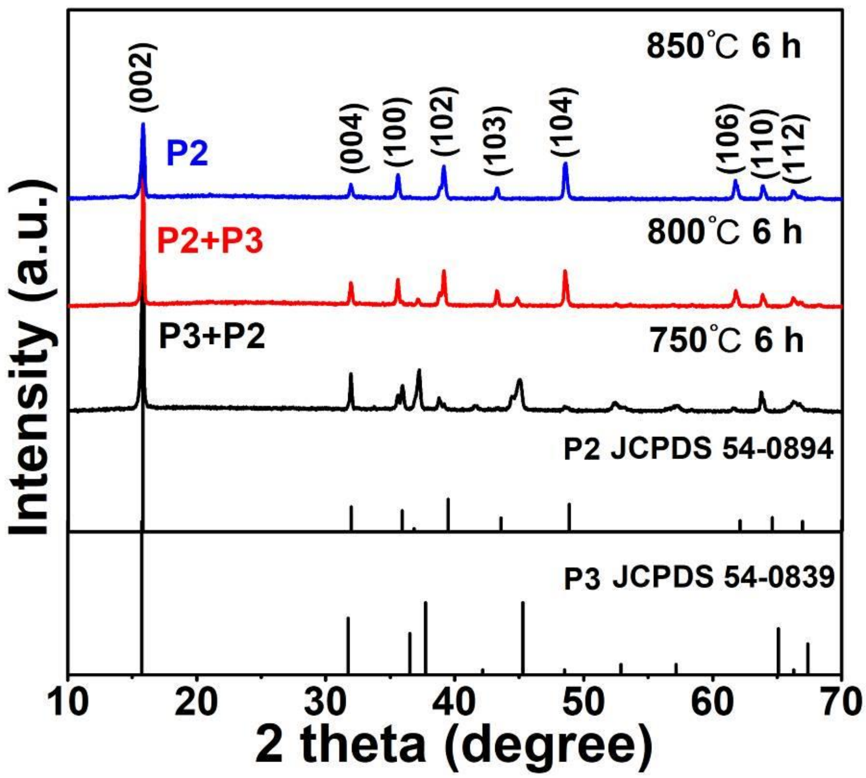

Figure S3. XRD patterns of the samples annealed at different temperatures for 6 h.

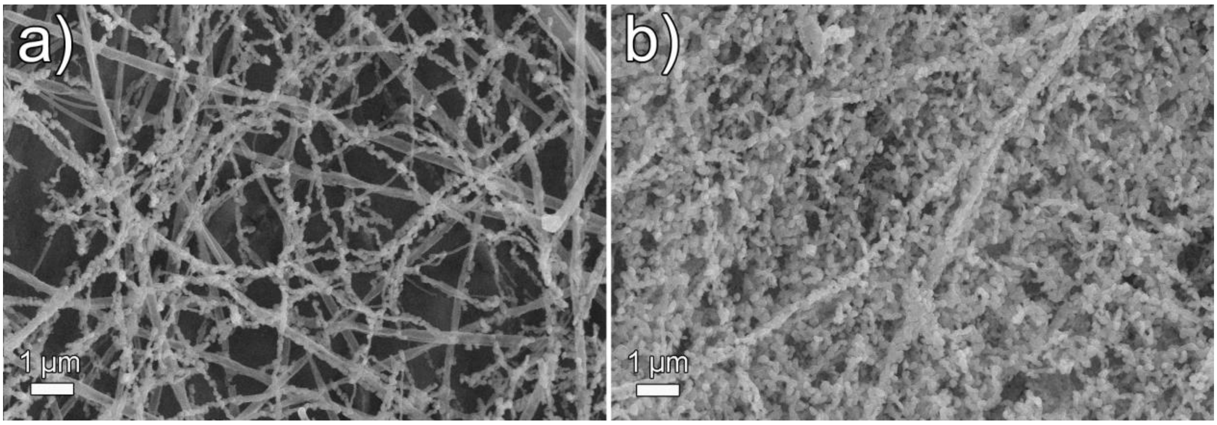

Figure S4. SEM images of the contrast samples after been heat treated at 850 °C for a) 2 h and b) 10 h, respectively.

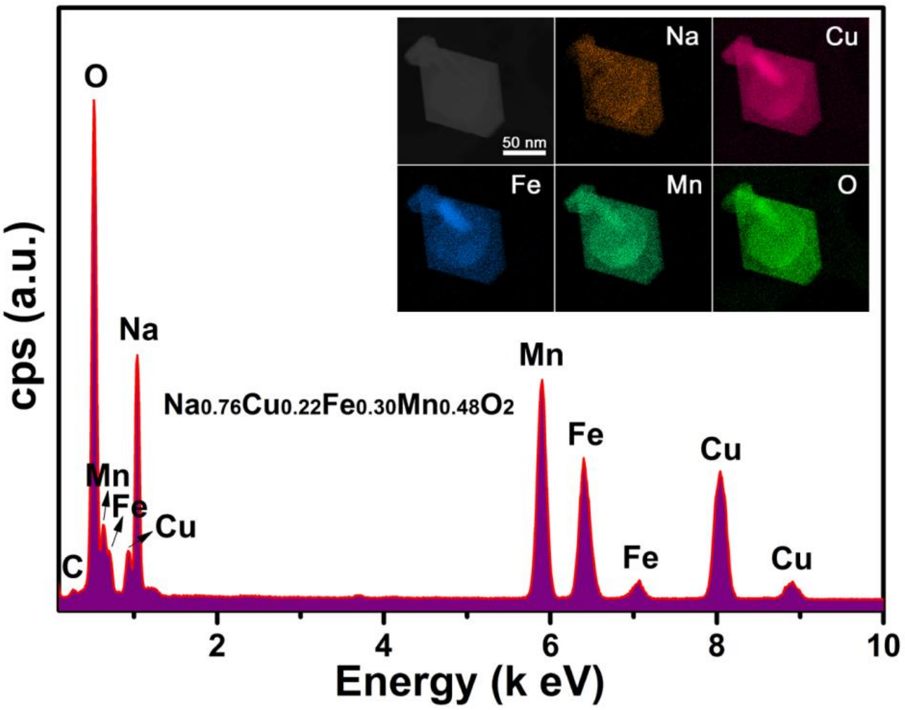

Figure S5. TEM energy dispersive spectroscopy (EDS) spectrum and mapping images (inset) of the P2-$Na_{0.76}Cu_{0.22}Fe_{0.30}Mn_{0.48}O_2$ nano-necklaces. The tiny carbon signal comes from the carbon support film.

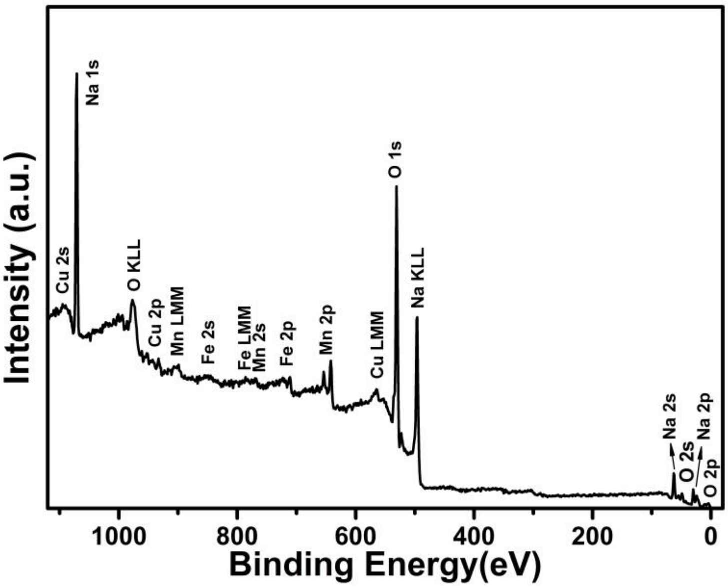

Figure S6. XPS survey spectrum of the P2-$Na_{0.76}Cu_{0.22}Fe_{0.30}Mn_{0.48}O_2$ nano-necklaces.

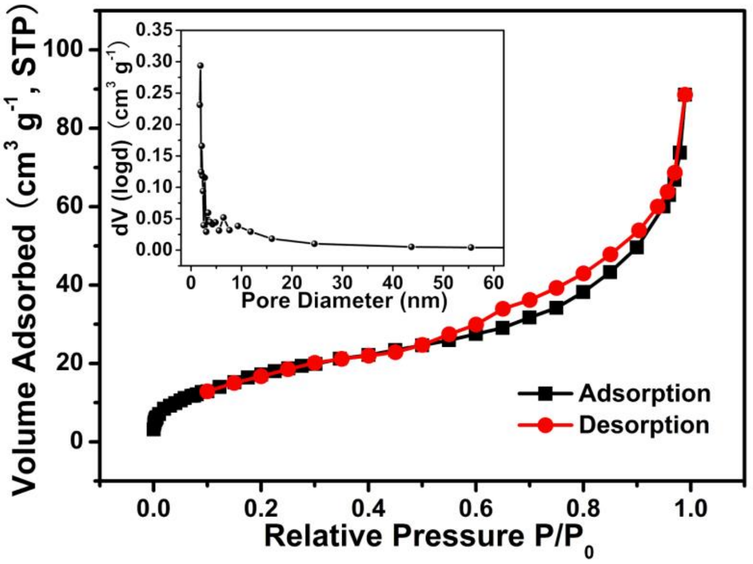

Figure S7. N_2 adsorption-desorption isotherms of the P2-$Na_{0.76}Cu_{0.22}Fe_{0.30}Mn_{0.48}O_2$ nano-necklaces. Inset is the pore size distribution curve.

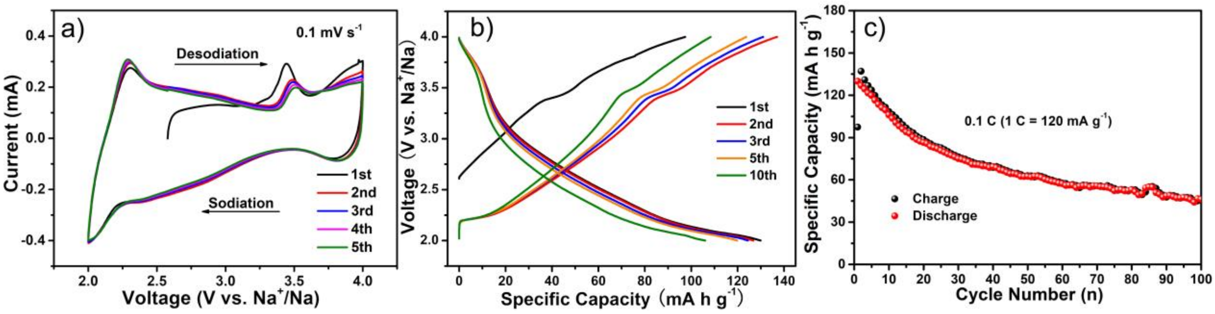

Figure S8. a) CV curves at a scan rate of 0.1 mV s^{-1}, b) galvanostatic charge/discharge profiles and c) cycling performance at 0.1 C (1 C = 120 mA g^{-1}) in the voltage window of 2.0-4.0 V vs. Na^+/Na of the P2-$Na_{2/3}Fe_{1/2}Mn_{1/2}O_2$ cathode.

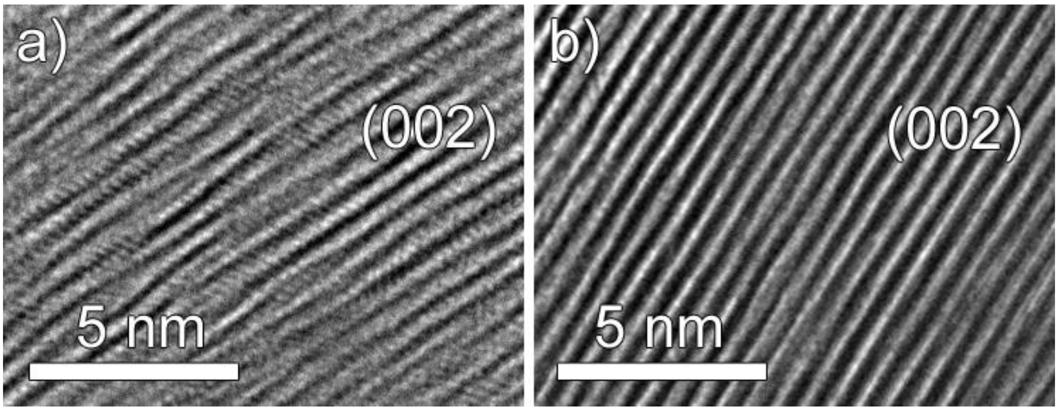

Figure S9. HRTEM images of the a) P2-$Na_{2/3}Fe_{1/2}Mn_{1/2}O_2$ and b) P2-$Na_{0.76}Cu_{0.22}Fe_{0.30}Mn_{0.48}O_2$ electrode materials after 10 cycles (current rate = 0.1 C).

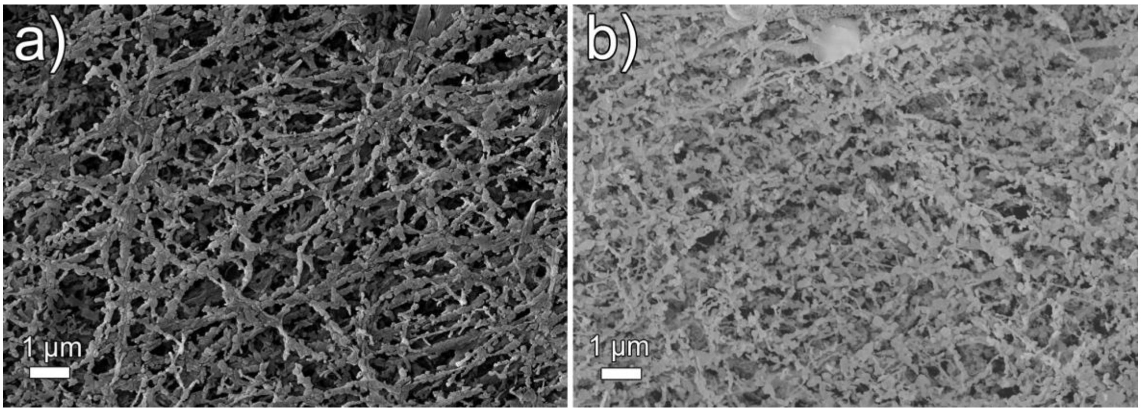

Figure S10. SEM images of a) the P2-$Na_{0.76}Cu_{0.22}Fe_{0.30}Mn_{0.48}O_2$ nano-necklaces electrode material and b) the contrast electrode material (annealed at 850 °C for 10 h) at the 4.0 V charged state after 100 cycles (current rate = 0.1 C).

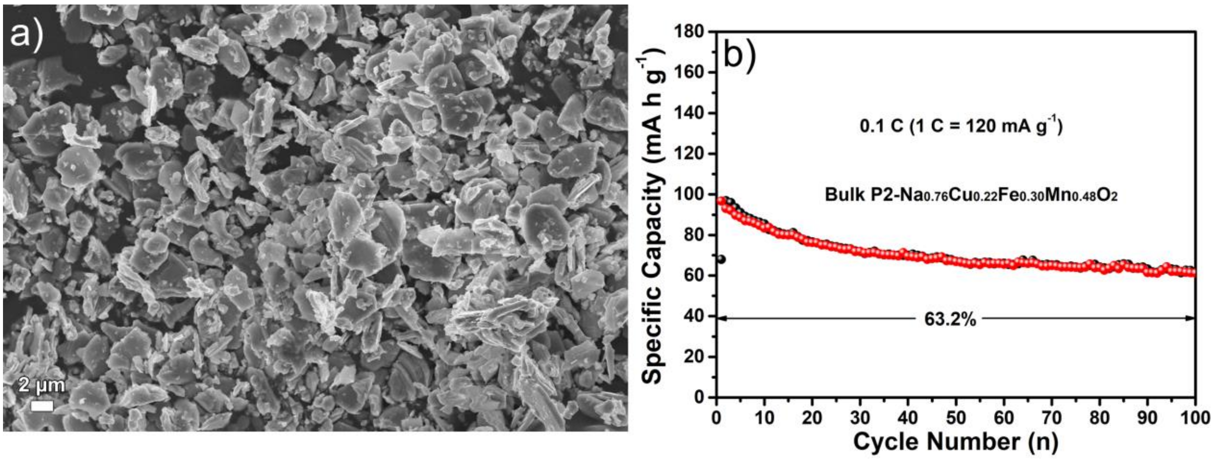

Figure S11. a) SEM image of the bulk P2-$Na_{0.76}Cu_{0.22}Fe_{0.30}Mn_{0.48}O_2$ sample, showing the flake-like particles with several microns in dimension, as well as b) the sodium-storage performance of the bulk P2-$Na_{0.76}Cu_{0.22}Fe_{0.30}Mn_{0.48}O_2$ electrode, delivering inferior reversible capacity and cycling performance (initial discharge capacity of 96.7 mA h g^{-1} with capacity retention of 63.2% after 100 cycles at 0.1 C) compared to those of the nano-necklaces cathode.

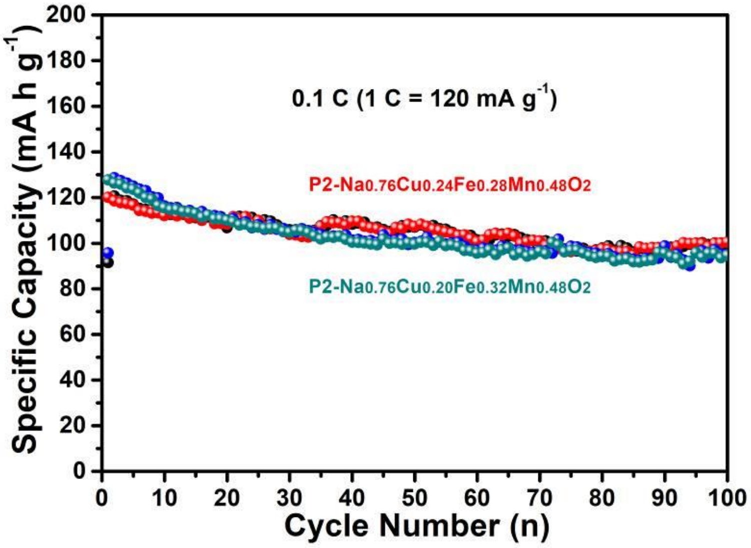

Figure S12. Cycling performance of the contrast P2-$Na_{0.76}Cu_{0.20}Fe_{0.32}Mn_{0.48}O_2$ (less Cu content) and P2-$Na_{0.76}Cu_{0.24}Fe_{0.28}Mn_{0.48}O_2$ (excess Cu content) nano-necklaces electrodes at 0.1 C.

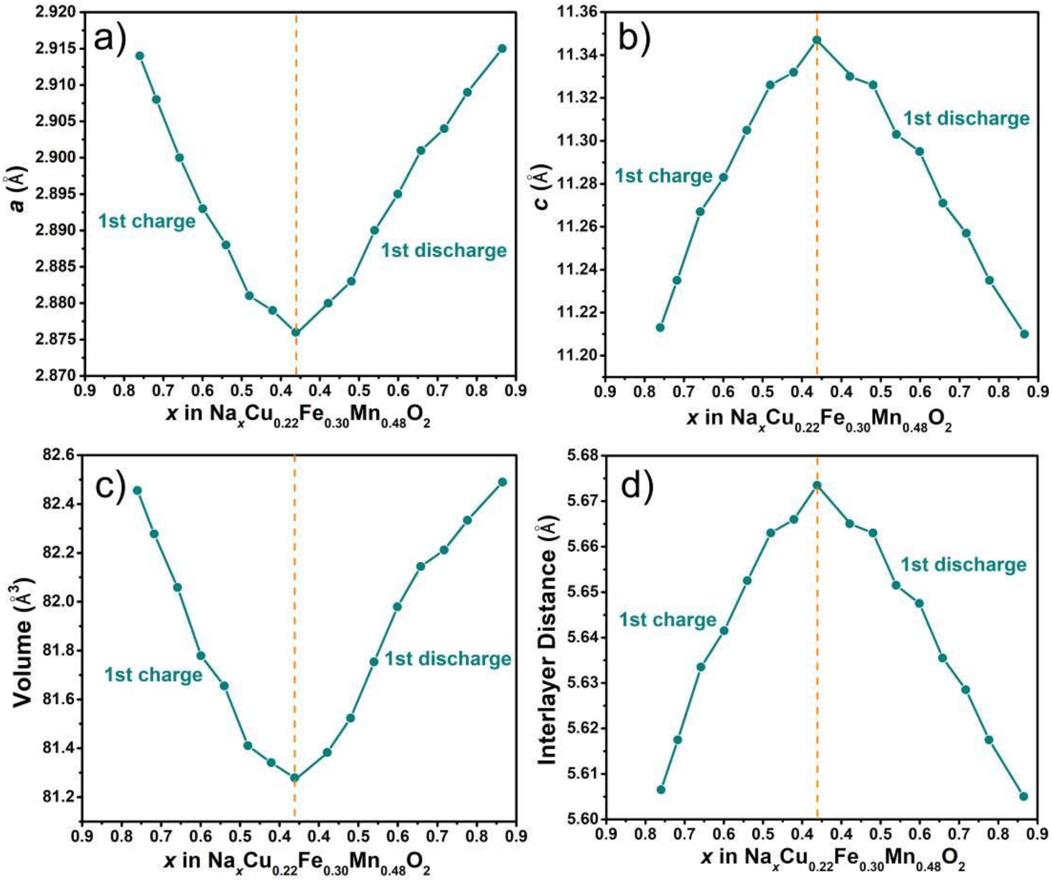

Figure S13. The evolutions of a) *a* and b) *c* parameters, c) cell volume, and d) the (002) interlayer distance upon charge/discharge during the first cycle.

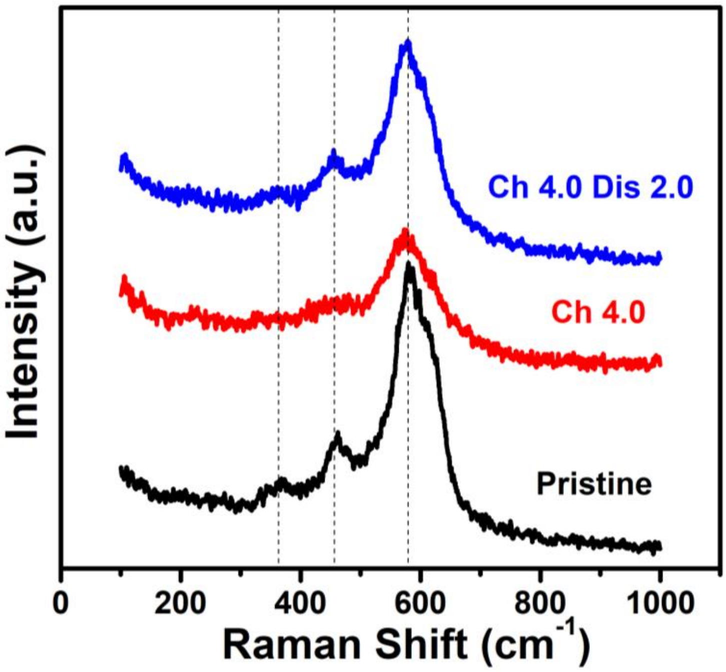

Figure S14. Ex situ Raman spectra of the P2-$Na_{0.76}Cu_{0.22}Fe_{0.30}Mn_{0.48}O_2$ nano-necklaces electrode at different charged/discharged states in the first cycle. The marked three bands are ascribed to the Na and O vibrations.[1,2]

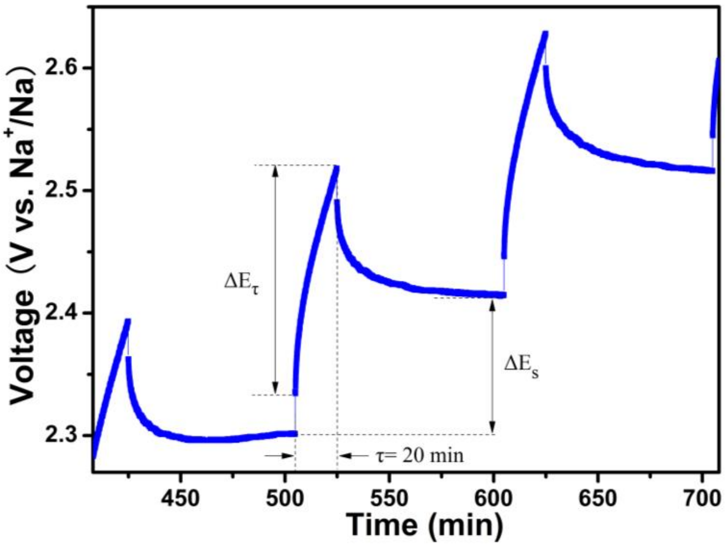

Figure S15. Schematic illustration of selected steps from the GITT profile during charging.

The D_{Na} can be calculated based on the simplified equation of $D_{Na} = \frac{4}{\pi\tau}\left(\frac{m_B V_M}{M_B A}\right)^2\left(\frac{\Delta E_S}{\Delta E_\tau}\right)^2$, where τ is the duration time of the current pulse, V_M is the molar volume (cm^3 mol^{-1}), m_B is the mass of the active material, M_B is the molecular weight (g mol^{-1}), A is the total contact area of electrode with electrolyte, ΔE_τ is the variation of the cell voltage, and ΔE_s is related to the change of steady-state voltage for the corresponding step.[2,3]

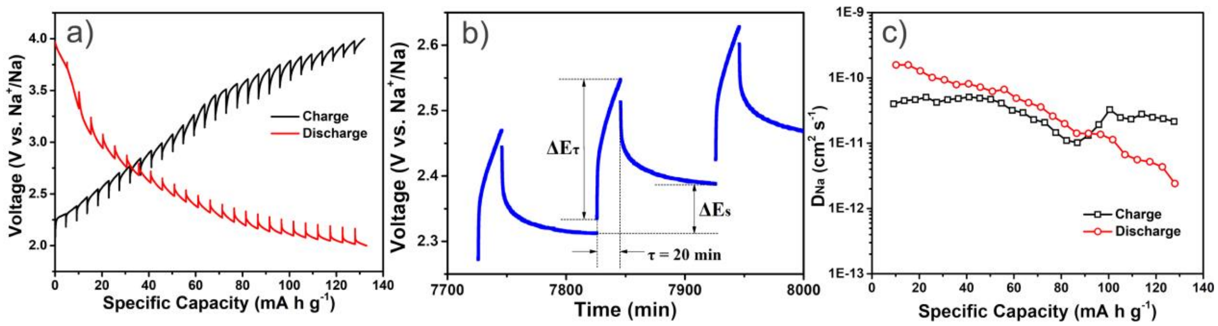

Figure S16. a) Charge-discharge GITT profiles of the P2-$Na_{2/3}Fe_{1/2}Mn_{1/2}O_2$ cathode at 15 mA g^{-1}, b) schematic illustration of selected steps from the GITT profile during charging, and c) the corresponding Na^+ diffusion coefficients (D_{Na}).

Figure S16a displays the typical discharge/charge GITT profile of the P2-$Na_{2/3}Fe_{1/2}Mn_{1/2}O_2$ cathode in the second cycle. The cell was discharged or charged at a constant current flux (15 mA g^{-1}) for an interval of 20 min and then relaxed for 80 min to allow the voltage reach equilibrium (as schematically shown in Figure S16b). This procedure was repeated for the full voltage window during battery operation. Accordingly, the D_{Na} values are calculated to be around 10^{-11} to 10^{-12} cm^2 s^{-1} (Figure S16c), based on the simplified equation of

$$D_{Na} = \frac{4}{\pi\tau}\left(\frac{m_B V_M}{M_B A}\right)^2 \left(\frac{\Delta E_S}{\Delta E_\tau}\right)^2 .$$

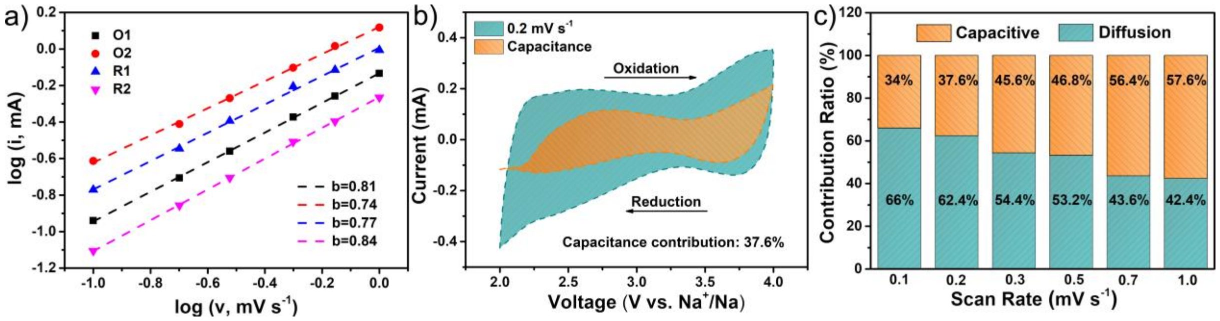

Figure S17. a) Linear relationships of logi_p versus log$v$, b) typical CV curve at 0.2 mV s^{-1} exhibiting the pseudo-capacitance contribution (orange region) to the total current, and c) percentages of the capacitive response and the diffusion-controlled intercalation in the charge storage process of the P2-$Na_{0.76}Cu_{0.22}Fe_{0.30}Mn_{0.48}O_2$ nano-necklaces cathode at different scan rates.

Generally speaking, both Faradaic intercalation and surface-controlled adsorption (pseudo-capacitance) exist in the charge storage process of the nanostructured electrode materials.[2] The capacitive behavior can be identified using the relation of $i_p = av^b$, in another form, logi_p = b × log$v$ + log$a$, where a and b are variable parameters. When the b value approaches 0.5, the redox reaction is dominated by ion diffusion; when the b value is close to 1.0, the reaction process relies on pseudo-capacitive behavior.[2,4] As revealed in Figure S17a, the b values for the O1, O2, R1, and R2 peaks are 0.81, 0.74, 0.77, and 0.84, respectively. This indicates that the diffusion-limited current and the capacitive response synergistically contribute to the electrode process, thus rendering the rapid reaction kinetics of the P2-$Na_{0.76}Cu_{0.22}Fe_{0.30}Mn_{0.48}O_2$ nano-necklaces cathode. Furthermore, the capacitive contribution can be quantitatively determined based on the equation of $i(V) = k_1v + k_2v^{1/2}$, where i(V) is the current at a fixed potential, while k_1v and $k_2v^{1/2}$ represent the capacitive effect and diffusion-controlled intercalation, respectively.[5] Figure S17b shows the representative CV profile at 0.2 mV s^{-1}, illustrating a 37.6% pseudo-capacitance contribution (orange area) to the total current. The capacitive effect gradually strengthens as the scan rate increases (Figure S17c).

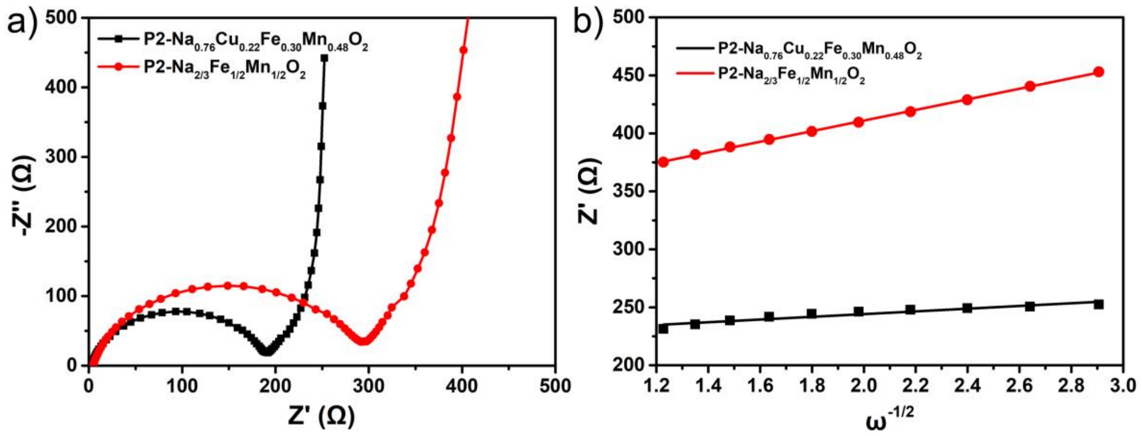

Figure S18. a) Nyquist plots of the P2-$Na_{0.76}Cu_{0.22}Fe_{0.30}Mn_{0.48}O_2$ and P2-$Na_{2/3}Fe_{1/2}Mn_{1/2}O_2$ electrodes, as well as b) the corresponding real parts of the impedance (Z′) versus reciprocal square root of the angular frequency (ω) in the low frequency region.

As shown in Figure S18a, the charge-transfer resistance (R_{ct}) of P2-$Na_{0.76}Cu_{0.22}Fe_{0.30}Mn_{0.48}O_2$ (~180 Ω) is smaller than that of P2-$Na_{2/3}Fe_{1/2}Mn_{1/2}O_2$ (~290 Ω), indicating the enhanced electronic conductivity. Moreover, the slope of Z′ vs. $\omega^{-1/2}$ plot of P2-$Na_{0.76}Cu_{0.22}Fe_{0.30}Mn_{0.48}O_2$ is smaller than that of P2-$Na_{2/3}Fe_{1/2}Mn_{1/2}O_2$ (Figure S18b), reflecting the higher Na^+ diffusion coefficient after Cu substitution.

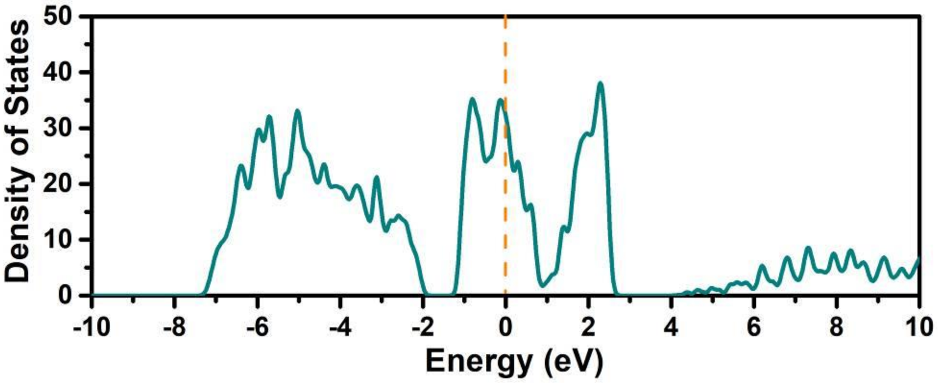

Figure S19. Density of states of the P2-$Na_{2/3}Fe_{1/2}Mn_{1/2}O_2$ cathode material.

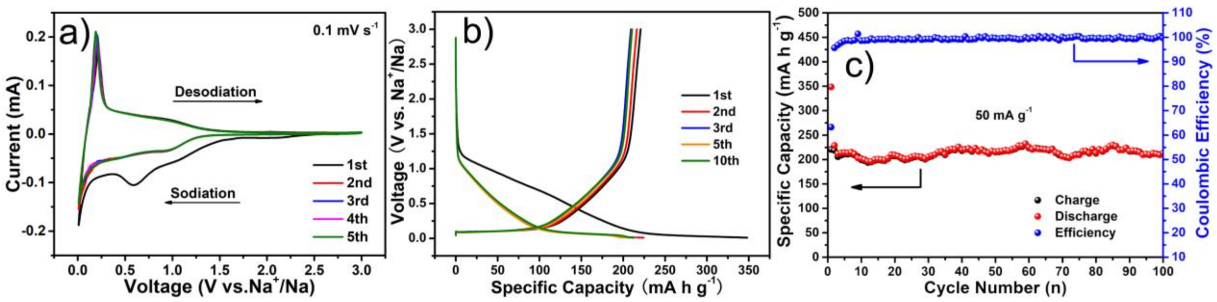

Figure S20. a) CV curves at a scan rate of 0.1 mV s^{-1}, b) galvanostatic charge/discharge profiles and c) cycling performance at a current density of 50 mA g^{-1} in the potential range of 0.01-3.0 V vs. Na^{+}/Na of the hard carbon anode.

Na-ion diffusion coefficients calculation based on EIS:

The apparent diffusion coefficient of Na^+ ions (D_{Na}) can be estimated from the low-frequency-region EIS spectrum according to the following equation (S1):

$$D_{Na} = \frac{R^2 T^2}{2 A^2 n^4 F^4 C^2 \sigma^2} \qquad \text{(S1)}$$

where R is the gas constant (8.314 J mol^{-1} K^{-1}), T is the absolute temperature (K), A is the contacting area of electrode with electrolyte (0.81 cm^2), n is the number of transferred electrons (n = 0.5 in this system), F is the Faraday constant (96500 C mol^{-1}), C is the concentration of sodium ions in the cathode (~3.06 × 10^{-2} mol cm^{-3}), and σ is the Warburg factor. The σ value can be determined from the slope of Z′ vs. $\omega^{-1/2}$ plot (Figure 4f) based on the equation (S2):[6,7]

$$Z' = R_s + R_{ct} + \sigma\omega^{-1/2} \qquad \text{(S2)}$$

As calculated, the σ of the P2-$Na_{0.76}Cu_{0.22}Fe_{0.30}Mn_{0.48}O_2$ nano-necklaces electrode (850 °C for 6 h) is the lowest, reflecting the highest D_{Na} of 6.747 × 10^{-12} cm^2 s^{-1}.

Table S1. Detailed structural information of P2-$Na_{0.76}Cu_{0.22}Fe_{0.30}Mn_{0.48}O_2$ determined from the Rietveld refined XRD pattern.

**Space group = $P6_3/mmc$**	**R_p = 5.48%**	**R_{wp} = 8.55%**
***a* (Å)= *b* (Å) = 2.9146**	***c* (Å) = 11.2104**	**V (Å^3) = 82.478**

**Atom**	**Wyckoff position**	***x***	***y***	***z***	**U_{iso}**	**Occupancy**
O	4f	0.3333	0.6666	0.0842	0.0681	1.0000
Na1	2b	0.0000	0.0000	0.2500	0.0554	0.2710
Na2	2d	0.3333	0.6666	0.7500	0.0412	0.4854
Cu	2a	0.0000	0.0000	0.0000	0.0519	0.2200
Fe	2a	0.0000	0.0000	0.0000	0.0519	0.3000
Mn	2a	0.0000	0.0000	0.0000	0.0519	0.4800

Table S2. Comparison of the electrochemical properties in this work with those previously reported for the Cu-doped Fe/Mn-based layered oxides Na-ion battery cathodes. (1 C = 120 mA g^{-1})

Sample	Particle Size	Rate Capability	Cyclic Stability	Voltage Range	Reference
**P2-$Na_{0.7}Cu_{0.15}Fe_{0.3}Mn_{0.55}O_2$**	/	97.8 mA h g^{-1} at 0.1 C<br>83.8 mA h g^{-1} at 0.2 C<br>75.1 mA h g^{-1} at 1 C<br>63.4 mA h g^{-1} at 2 C	82% capacity retention at 0.1 C after 80 cycles	2.5-4.2 V	[8]
**P2-$Na_{0.7}Cu_{0.2}Fe_{0.2}Mn_{0.6}O_2$**	/	82.2 mA h g^{-1} at 0.1 C<br>80.8 mA h g^{-1} at 0.2 C<br>78.2 mA h g^{-1} at 0.5 C<br>75.6 mA h g^{-1} at 1 C<br>60.8 mA h g^{-1} at 2 C	80% capacity retention at 0.2 C after 80 cycles	2.5-4.2 V	[8]
**P2-$Na_{7/9}Cu_{2/9}Fe_{1/9}Mn_{2/3}O_2$ Microflakes**	2-10 μm	90 mA h g^{-1} at 0.1 C<br>83 mA h g^{-1} at 0.2 C<br>71 mA h g^{-1} at 0.5 C<br>64 mA h g^{-1} at 1 C<br>51 mA h g^{-1} at 2 C	87% capacity retention at 0.1 C after 150 cycles	2.5-4.2 V	[9]
**O3-$Na_{0.9}Cu_{0.22}Fe_{0.30}Mn_{0.48}O_2$ Microflakes**	3 μm	98 mA h g^{-1} at 0.1 C<br>94 mA h g^{-1} at 0.2 C<br>88 mA h g^{-1} at 0.5 C<br>78 mA h g^{-1} at 1 C<br>72 mA h g^{-1} at 2 C<br>59 mA h g^{-1} at 5 C	97% capacity retention at 0.1 C after 100 cycles	2.5-4.05 V	[10]
**Plate-like P2-$Na_{0.67}Mn_{0.66}Fe_{0.20}Cu_{0.14}O_2$**	1-3 μm	94 mA h g^{-1} at 0.05 C<br>87.4 mA h g^{-1} at 0.1 C<br>77.7 mA h g^{-1} at 0.2 C	84% capacity retention at 0.05 C after 100 cycles	2.1-4.1 V	[11]
**Round-shaped O3-$Na[Cu_{0.2}(Fe_{1/3}Mn_{2/3})_{0.8}]O_2$ particles**	2-3 μm	131 mA h g^{-1} at 0.1 C<br>124 mA h g^{-1} at 0.2 C<br>102 mA h g^{-1} at 0.5 C<br>87 mA h g^{-1} at 1 C<br>70 mA h g^{-1} at 2 C	70% capacity retention at 2 C after 30 cycles	1.2-4.3 V	[12]

**O3-$Na_{0.9}Cu_{1/4}Fe_{1/4}Mn_{1/4}Ti_{1/4}O_2$ Microparticles**	2-5 µm	94 mA h g^{-1} at 0.1 C	60% capacity retention at 1 C after 90 cycles	2.5-4.0 V	[13]
**O3-$Na_{0.91}Ca_{0.02}[Cu_{0.24}Fe_{0.2}Mn_{0.56}]S_{0.05}O_{2-\sigma}$**	0.5-5 µm	107.6 mA h g^{-1} at 0.1 C<br>105 mA h g^{-1} at 0.2 C<br>92 mA h g^{-1} at 0.5 C<br>85 mA h g^{-1} at 1 C<br>78 mA h g^{-1} at 2 C<br>58 mA h g^{-1} at 5 C	84% capacity retention at 2 C after 200 cycles	2.2-4.2 V	[14]
**P2-$Na_{0.76}Cu_{0.22}Fe_{0.30}Mn_{0.48}O_2$ Nano-Necklaces**	50-150 nm	125.4 mA h g^{-1} at 0.1 C<br>112 mA h g^{-1} at 0.2 C<br>99.8 mA h g^{-1} at 0.5 C<br>92.9 mA h g^{-1} at 1 C<br>79 mA h g^{-1} at 2 C<br>73.9 mA h g^{-1} at 5 C<br>65 mA h g^{-1} at 10 C<br>56.5 mA h g^{-1} at 20 C	82.7% capacity retention at 0.1 C after 100 cycles<br>79% capacity retention at 2 C after 300 cycles	2.0-4.0 V	**This work**

Supplementary References

[1] P. Wang, Y. You, Y. X. Yin, Y. Wang, L. Wan, L. Gu, Y.-G, Guo, *Angew. Chem. Int. Ed.* **2016**, *55*, 7445-7449.

[2] Y. C. Liu, Q. Shen, X. Zhao, J. Zhang, X. Liu, T. Wang, N. Zhang, L. F. Jiao, J. Chen, L.-Z. Fan, *Adv. Funct. Mater.* **2020**, *30*, 1907837.

[3] N. Zhang, M. Jia, Y. Dong, Y. Wang, J. Xu, Y. C. Liu, L. F. Jiao, F. Y. Cheng, *Adv. Funct. Mater.* **2019**, *29*, 1807331.

[4] V. Augustyn, J. Come, M. A. Lowe, J. W. Kim, P. L. Taberna, S. H. Tolbert, H. D. Abruna, P. Simon, B. Dunn, *Nat. Mater.* **2013**, *12*, 518.

[5] D. L. Chao, P. Liang, Z. Chen, L. Bai, H. Shen, X. Liu, X. H. Xia, Y. Zhao, S. V. Savilov, J. Lin, Z. X. Shen, *ACS Nano* **2016**, *10*, 10211.

[6] Y. C. Liu, N. Zhang, L. F. Jiao, J. Chen, *Adv. Mater.* **2015**, *27*, 6702-6707.

[7] B. H. Li, C. Han, Y. B. He, C. Yang, H. Du, Q. H. Yang, F. Y. Kang, *Energy Environ. Sci.* **2012**, *5*, 9595-9602.

[8] S. Xu, J. Wu, E. Hu, Q. Li, J. Zhang, Y. Wang, E. Stavitski, L. Jiang, X. Rong, X. Yu, W. Yang, X. Yang, L. Q. Chen, Y.-S. Hu, *J. Mater. Chem. A* **2018**, *6*, 20795-20803.

[9] Y. Li, Z. Yang, S. Xu, L. Mu, L. Gu, Y.-S. Hu, H. Li, L. Q. Chen, *Adv. Sci.* **2015**, *2*, 1500031.

[10] L. Mu, S. Xu, Y. Li, Y.-S. Hu, H. Li, L. Q. Chen, X. J. Huang, *Adv. Mater.* **2015**, *27*, 6928-6933.

[11] E. S. Talaie, Y. Kim, N. Chen, L. F. Nazar, *Chem. Mater.* **2017**, *29*, 6684-6697.

[12] S. M. Oh, P. Oh, S. O. Kim, A. A. Manthiram, *J. Electrochem. Soc.* **2017**, *164*, A321-A326.

[13] L. Mu, Y.-S. Hu, L. Q. Chen, *Chin. Phys. B* **2015**, *24*, 038202.

[14] X. Gao, F. Jiang, Y. Yang, Y. Zhang, G. Zou, H. Hou, Y. Hu, W. Sun, X. B. Ji, *ACS Appl. Mater. Interfaces* **2020**, *12*, 2432-2444.
